# Supplementary material for: 7-Chloroquinolinehydrazones as First-in-Class Anticancer Experimental Drugs in the NCI-60 Screen among Different Investigated Series of Aryl, Quinoline, Pyridine, Benzothiazole and Imidazolehydrazones
Source: Pharmaceuticals (Basel). 2023 May 3;16(5):691. doi: 10.3390/ph16050691 (PMC10221356; doi:10.3390/ph16050691)
Supplement: Supplementary file 1 [file pharmaceuticals-16-00691-s001.zip › pharmaceuticals-2368525-supplementary.pdf]

7-Chloroquinolinehydrazones as First-in-Class Anticancer Experimental Drugs in the NCI-60 Screen among Different Investigated Series of Aryl, Quinoline, Pyridine, Benzothiazole and Imidazolehydrazones

Georgiana Negru (Apostol) [1], Alina Ghinet [1,2,3,*] and Elena Bîcu [1,*]

1 Faculty of Chemistry, 'Alexandru Ioan Cuza' University of Iasi, Bd. Carol I, nr. 11, 700506 Iasi, Romania; georgiana_16_95@yahoo.com
2 Junia, Health and Environment, Laboratory of Sustainable Chemistry and Health, F-59000 Lille, France
3 Institut National de la Santé et de la Recherche Médicale, Centre Hospitalier Universitaire de Lille, Institut Pasteur Lille, U1167—RID-AGE—Facteurs de Risque et Déterminants Moléculaires des Maladies Liées au Vieillissement, University of Lille, F-59000 Lille, France
* Correspondence: alina.ghinet@junia.com (A.G.); elena@uaic.ro (E.B.)

-Supplementary material-

1-(3-Bromophenyl)-2-(3,4,5-trimethoxybenzylidene)hydrazine (**1**).

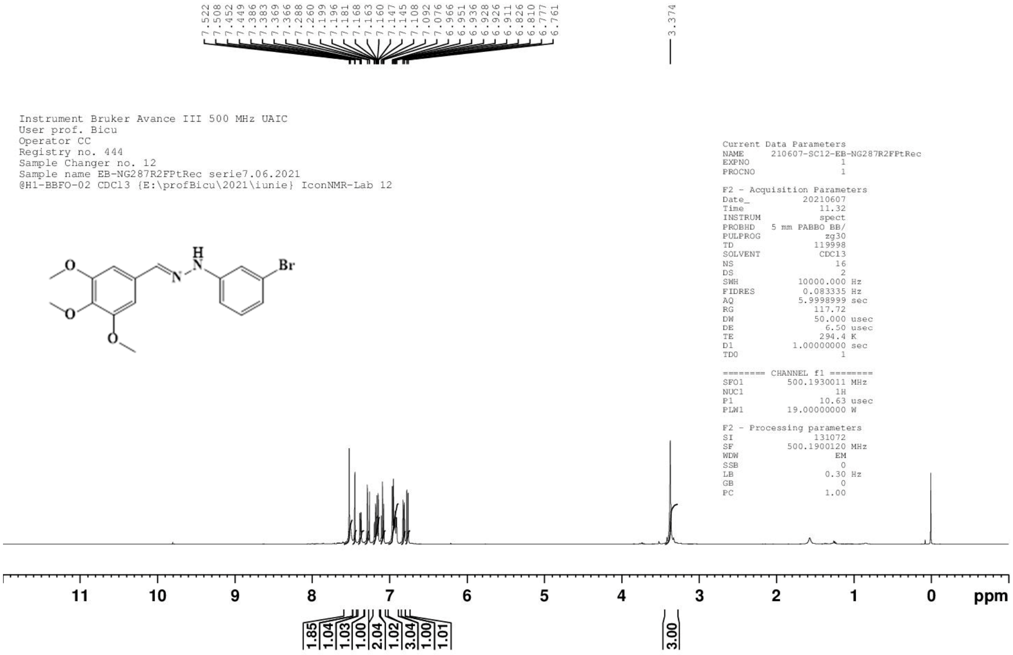

(a)

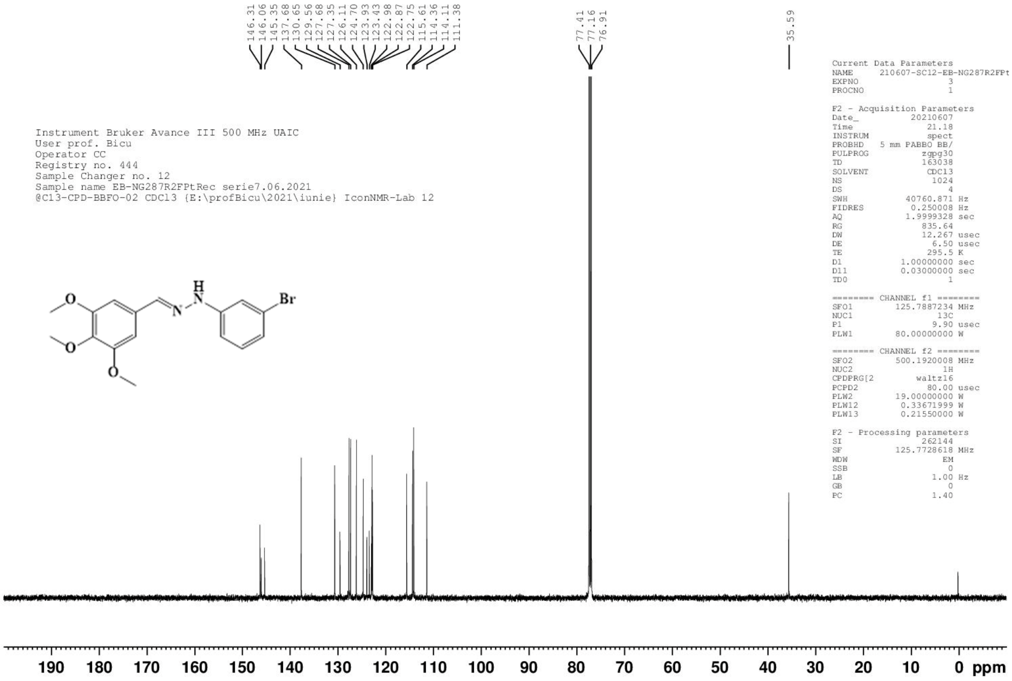

(b)

Figure S1. (a) ^{1}H-NMR and (b) ^{13}C-NMR spectra for hydrazone **1**

1-(3,4-dimethylphenyl)-2-(4-nitrobenzylidene)hydrazine (**2**).

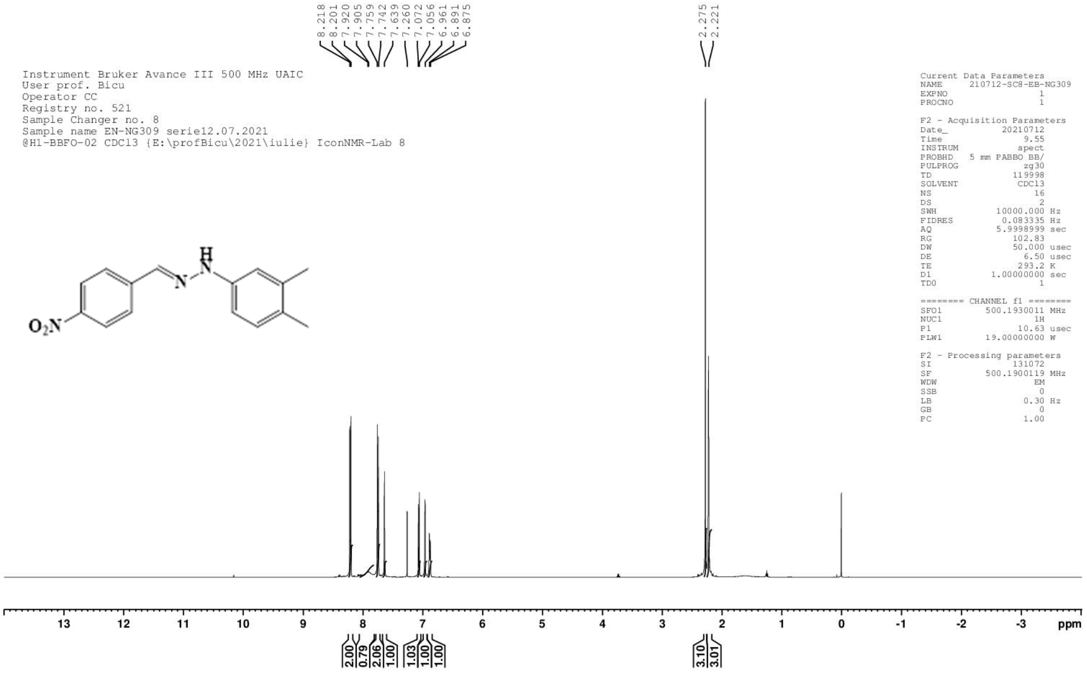

(a)

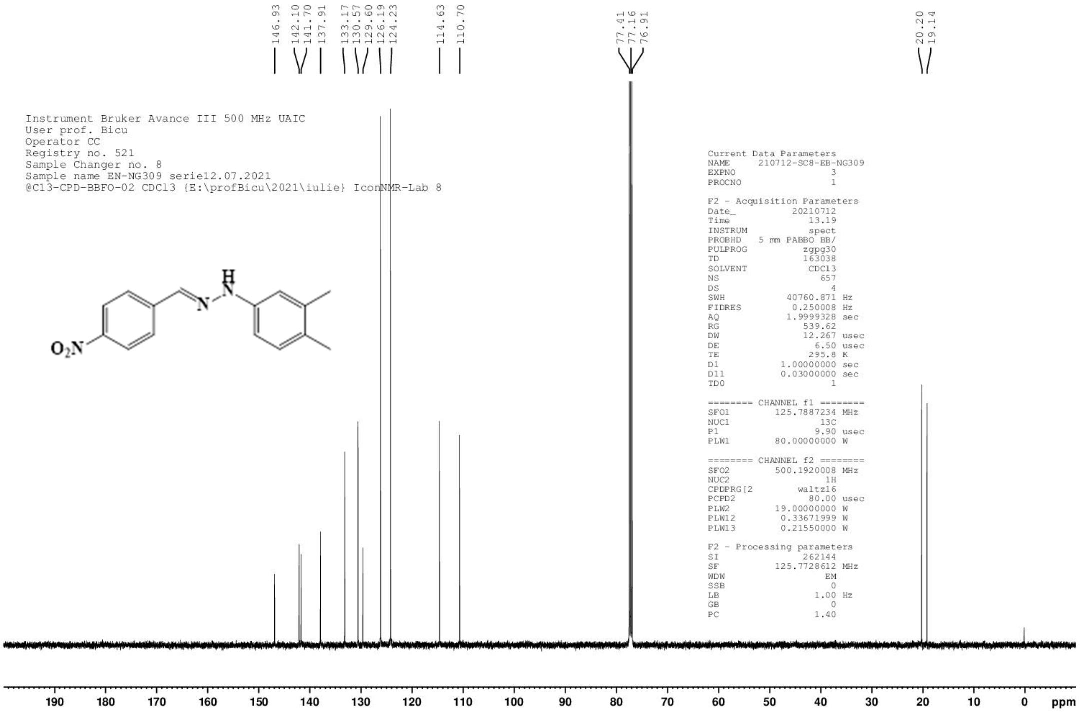

(b)

Figure S2. (a) ^{1}H-NMR and (b) ^{13}C-NMR spectra for hydrazone **2**

2-(2-(4-nitrobenzylidene)hydrazinyl)-4,5-dihydro-1*H*-imidazole (**3**)

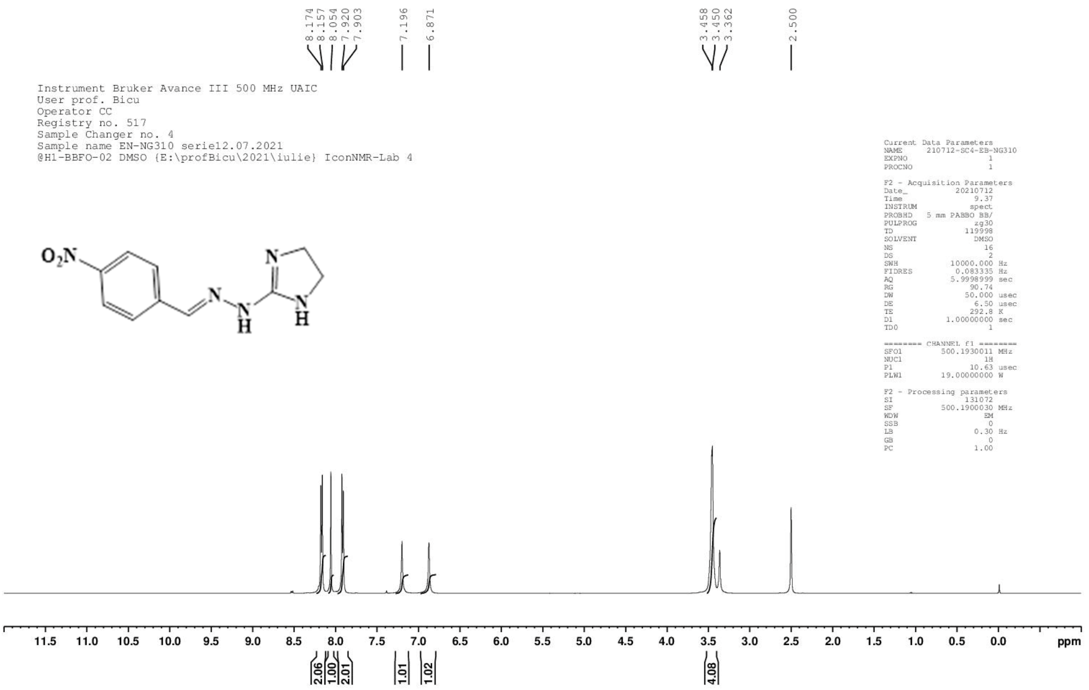

(a)

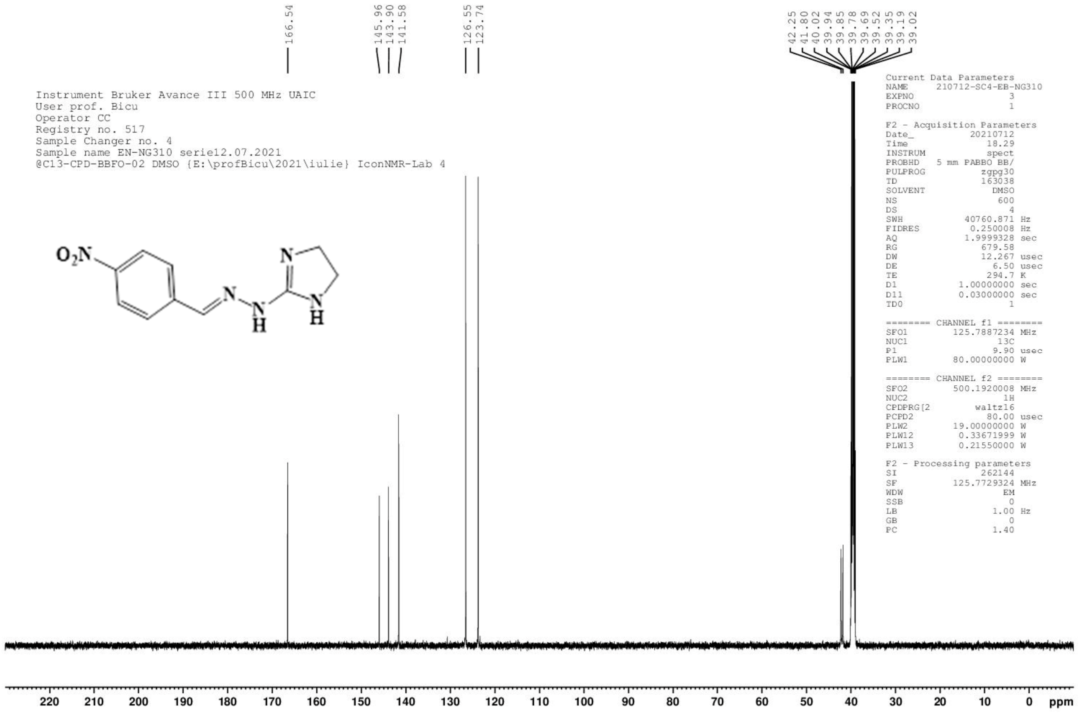

(b)

Figure S3. (a) ^{1}H-NMR and (b) ^{13}C-NMR spectra for hydrazone **3**

2-(2-(thiophen-2-ylmethylene)hydrazinyl)-4,5-dihydro-1*H*-imidazole (**4**).

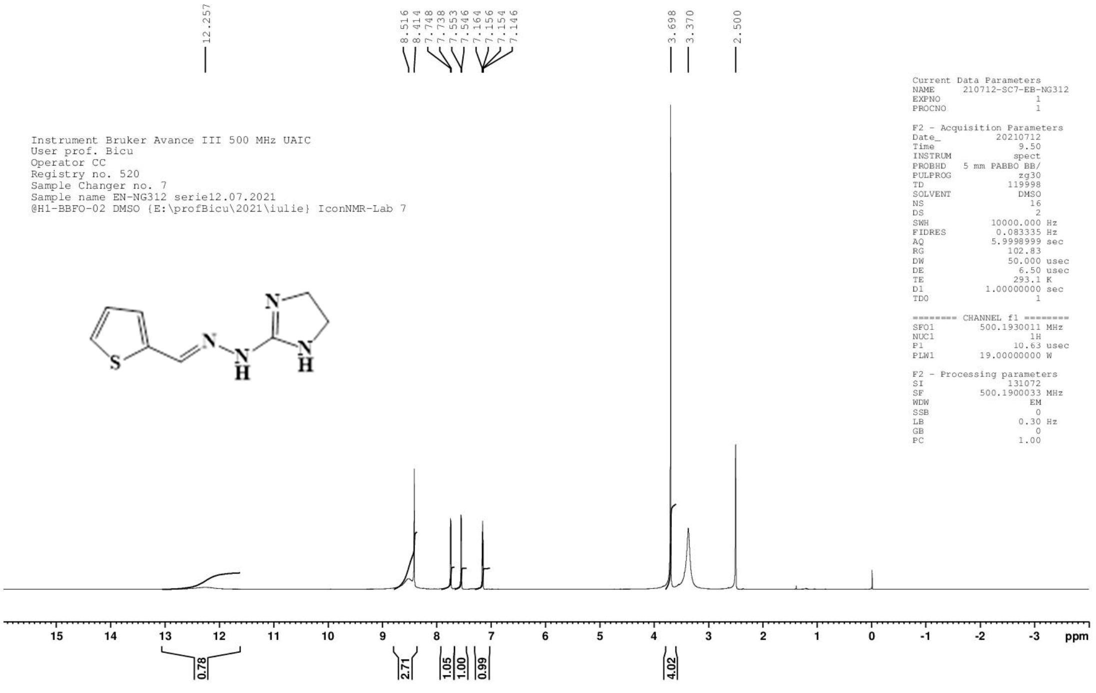

(a)

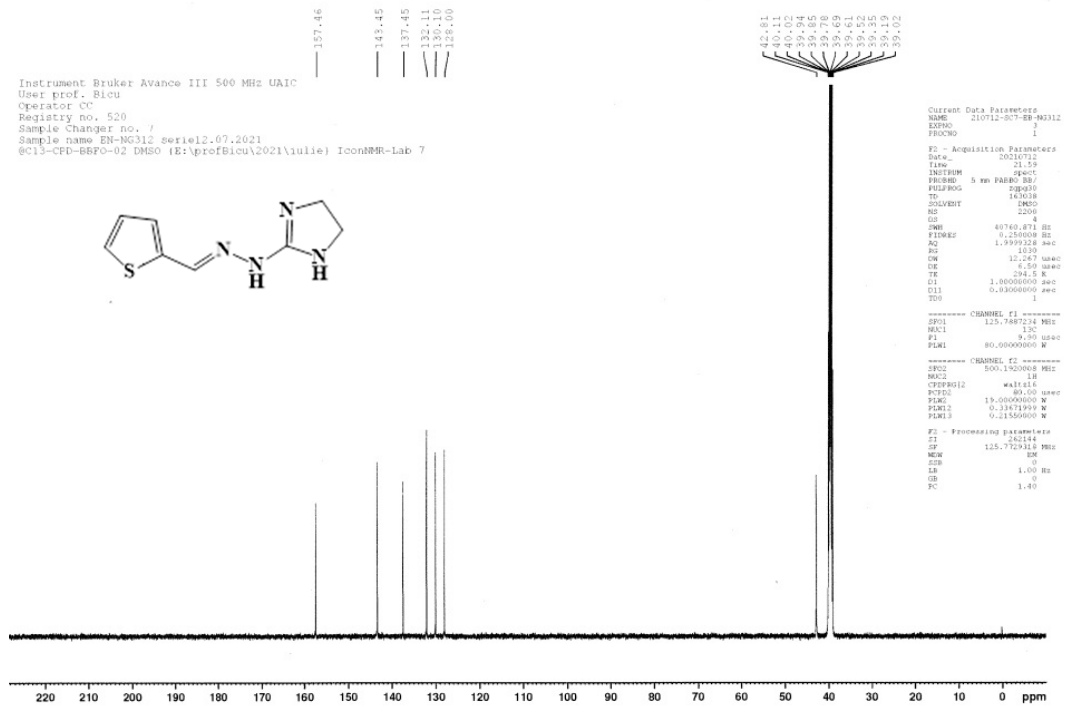

(b)

Figure S4. (a) ^{1}H-NMR and (b) ^{13}C-NMR spectra for hydrazone **4**

2-(2-(2,4-dichlorobenzylidene)hydrazinyl)-4,5-dihydro-1*H*-imidazole (**5**).

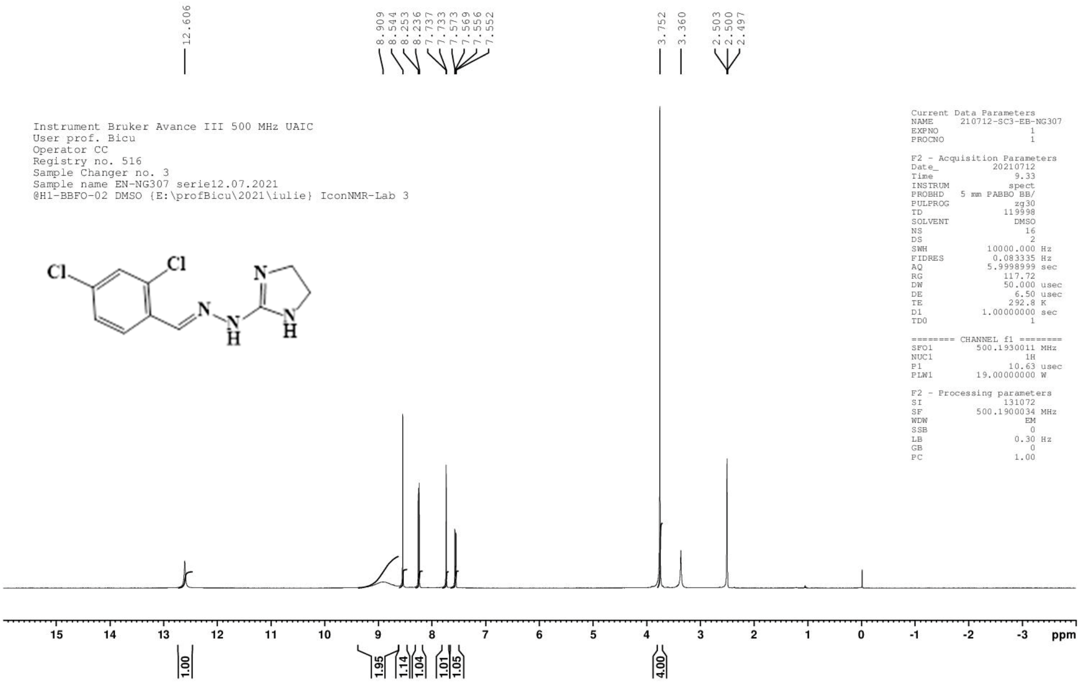

(a)

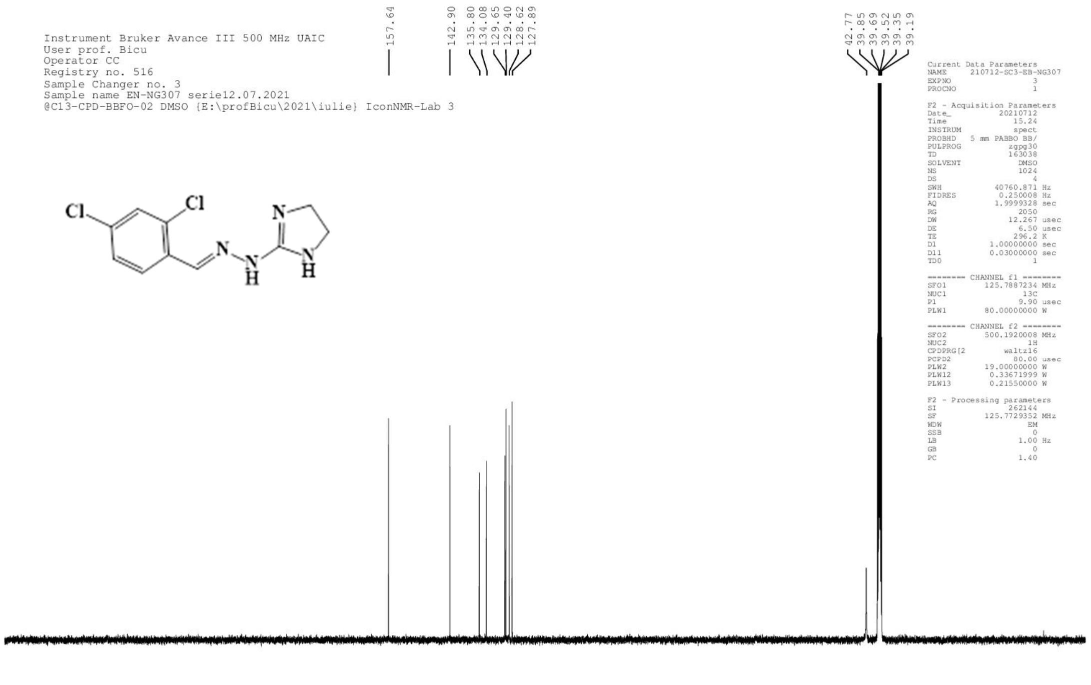

(b)

Figure S5. (a) ^{1}H-NMR and (b) ^{13}C-NMR spectra for hydrazone **5**

7-chloro-4-(2-(3-chloro-4-methoxybenzylidene)hydrazinyl)quinoline (**6**).

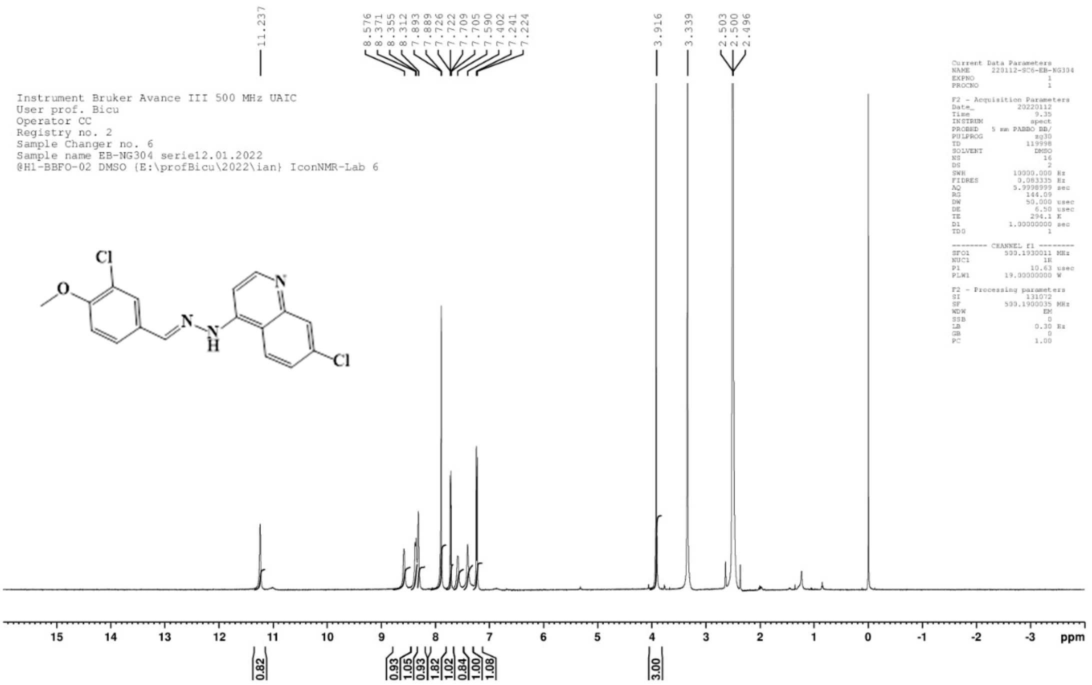

(a)

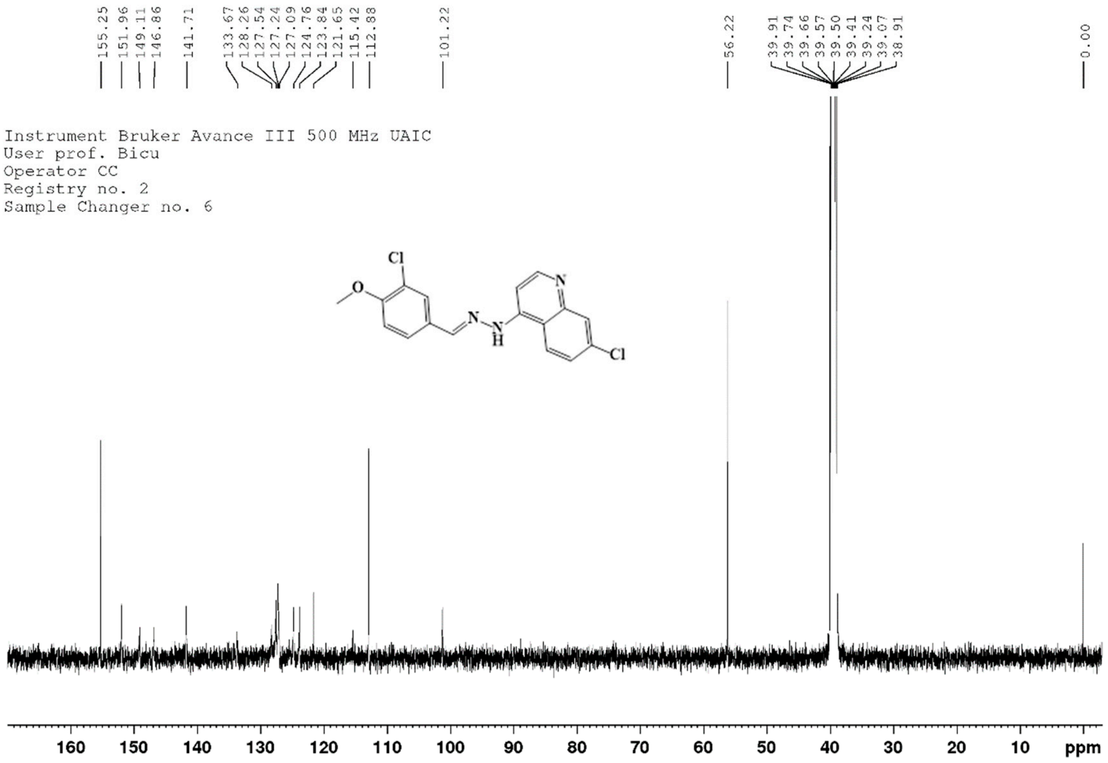

(b)

Figure S6. (a) ^{1}H-NMR and (b) ^{13}C-NMR spectra for hydrazone **6**

4-((2-(7-chloroquinolin-4-yl)hydrazono)methyl)-2,6-dimethoxyphenol (**7**).

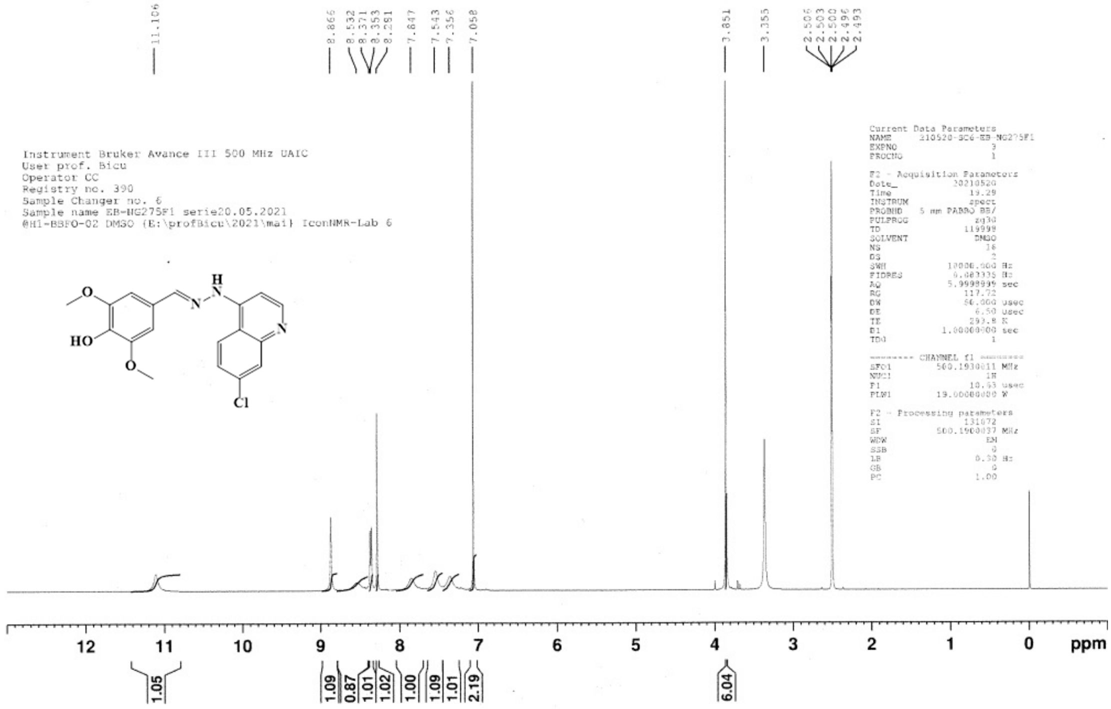

(a)

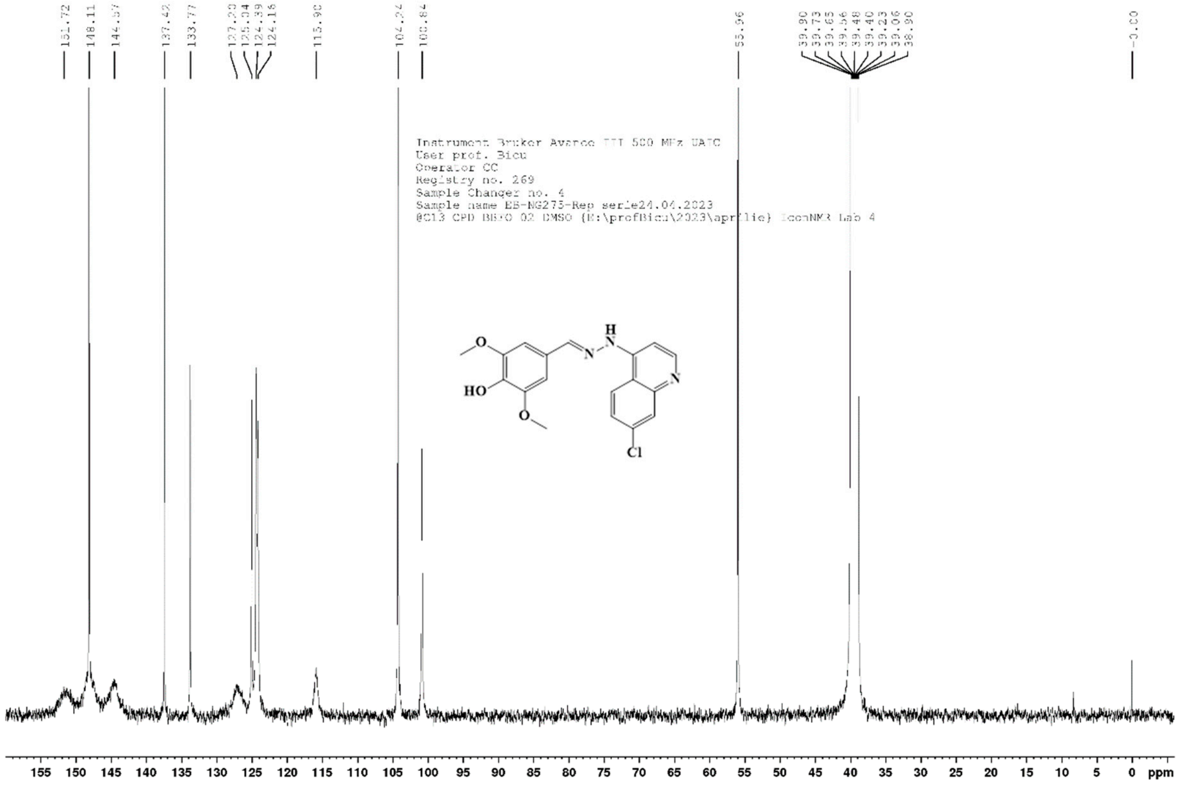

(b)

Figure S7. (a) ^{1}H-NMR and (b) ^{13}C-NMR spectra for hydrazone **7**

3-((2-(7-chloroquinolin-4-yl)hydrazono)methyl)-10-methyl-10*H*-phenothiazine (**8**).

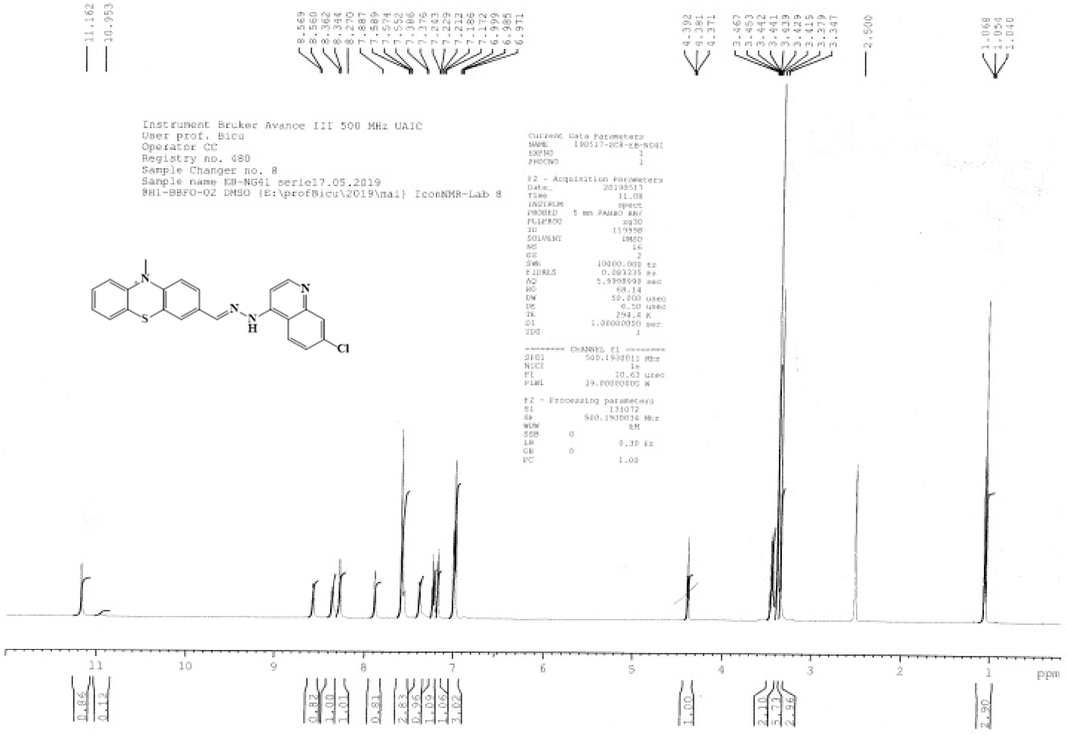

(a)

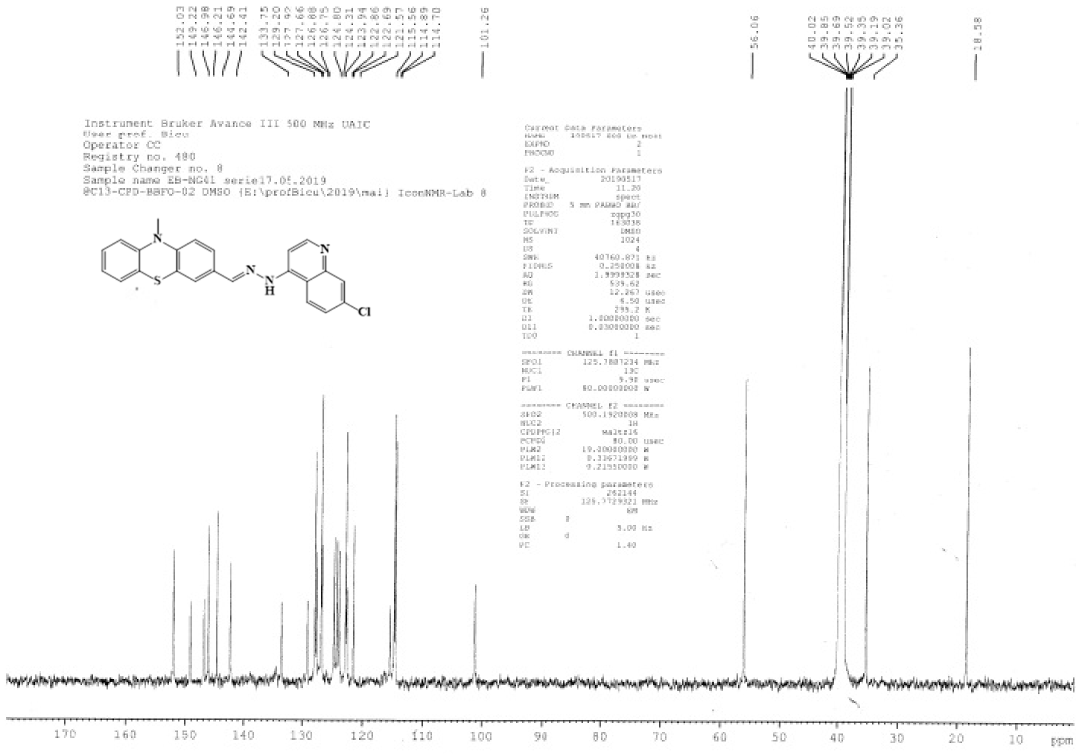

(b)

Figure S8. (a) ^{1}H-NMR and (b) ^{13}C-NMR spectra for hydrazone **8**

3-((2-(4-methoxyphenyl)hydrazono)methyl)-10-methyl-10*H*-phenothiazine (**9**).

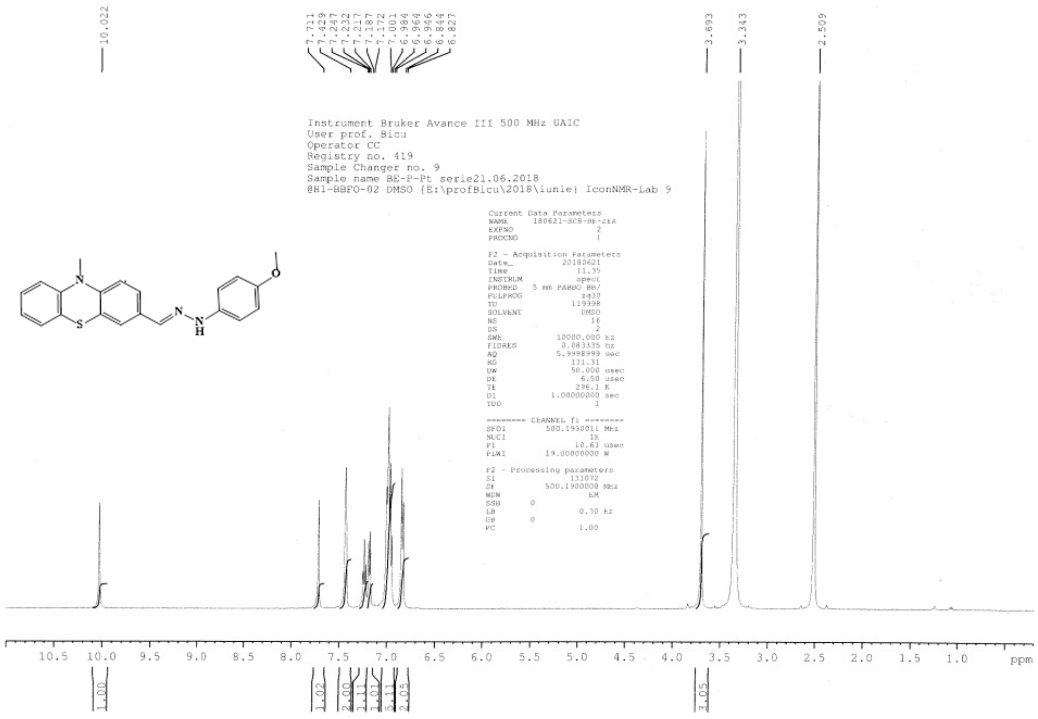

(a)

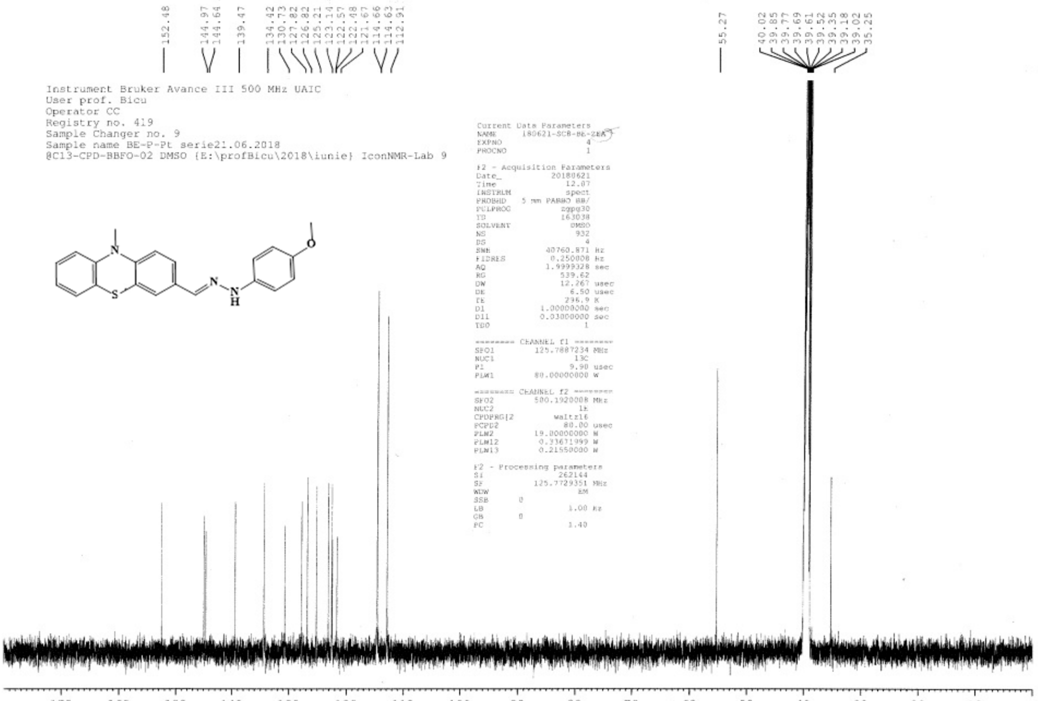

(b)

Figure S9. (a) ^{1}H-NMR and (b) ^{13}C-NMR spectra for hydrazone **9**

3-((2-(benzo[*d*]thiazol-2-yl)hydrazono)methyl)-10-methyl-10*H*-phenothiazine (**10**).

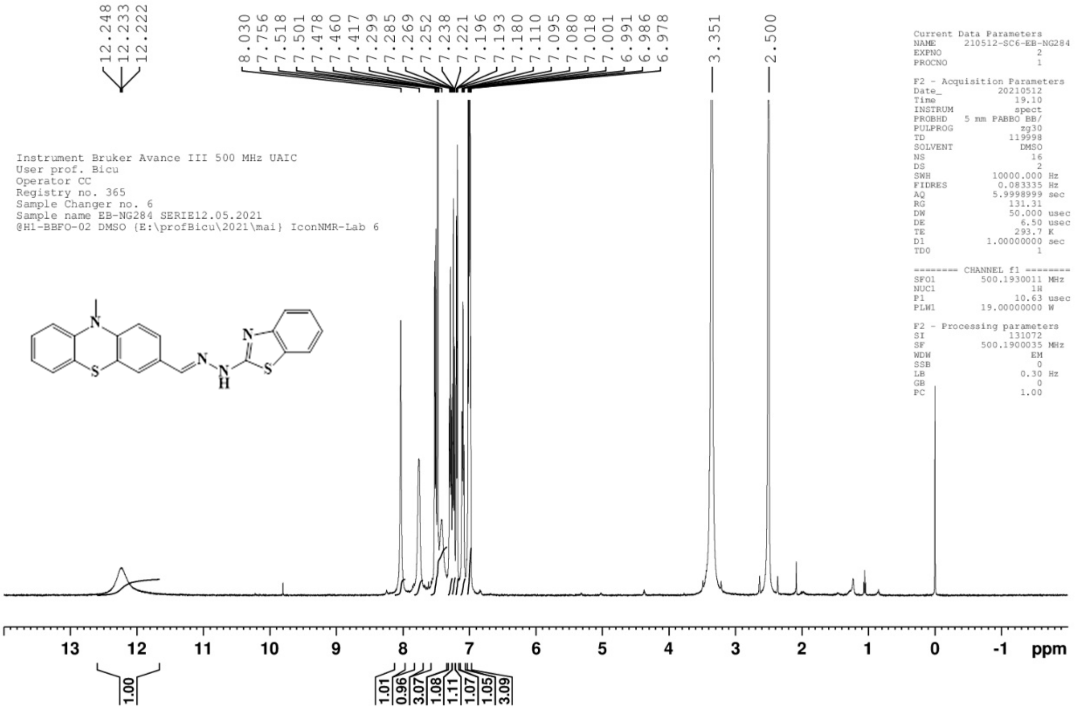

(a)

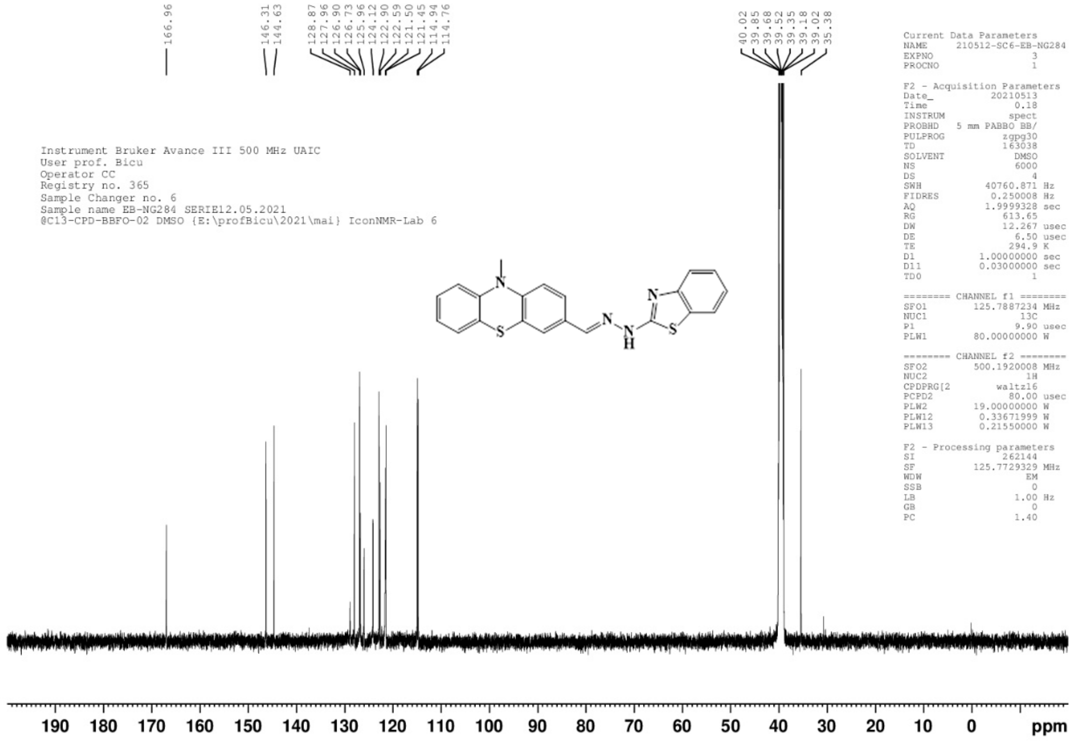

(b)

Figure S10. (a) ^{1}H-NMR and (b) ^{13}C-NMR spectra for hydrazone **10**

3-((2-(4-bromophenyl)hydrazono)methyl)-10-methyl-10*H*-phenothiazine (**11**).

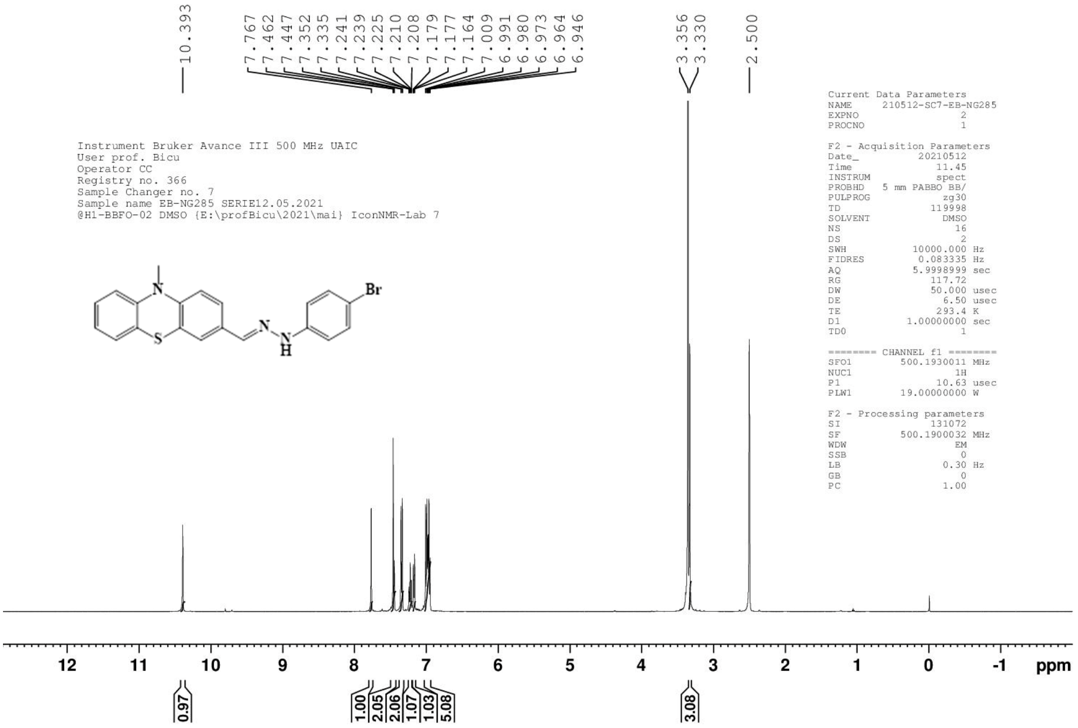

(a)

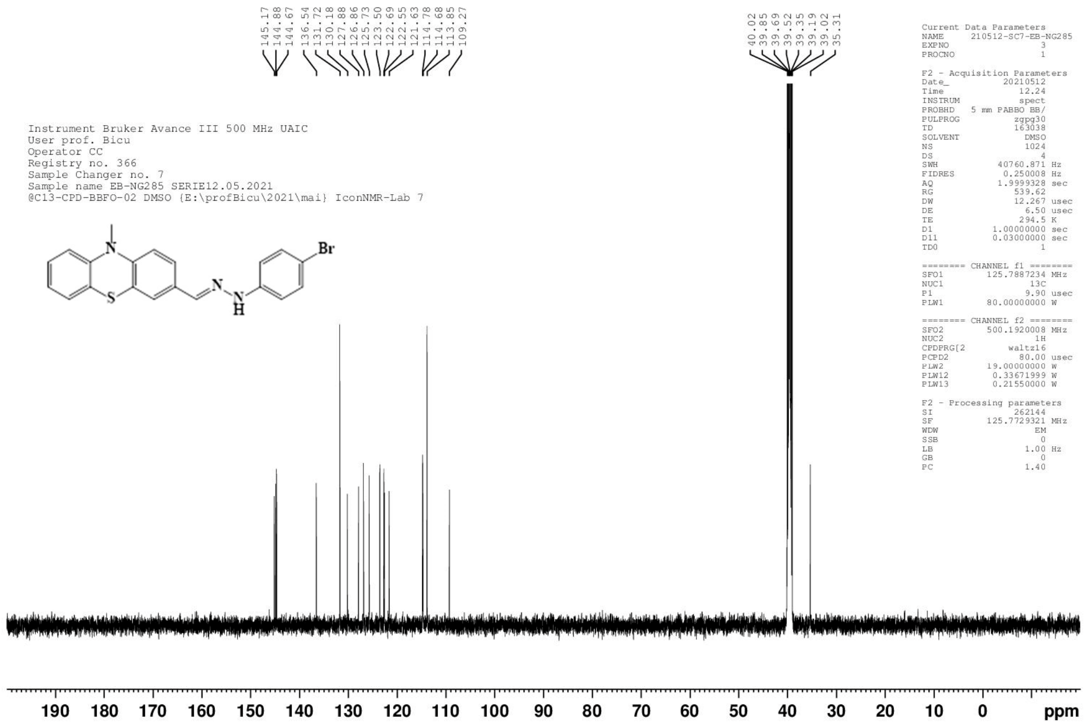

(b)

Figure S11. (a) ^{1}H-NMR and (b) ^{13}C-NMR spectra for hydrazone **11**

3-((2-(3-bromophenyl)hydrazono)methyl)-10-methyl-10*H*-phenothiazine (**12**).

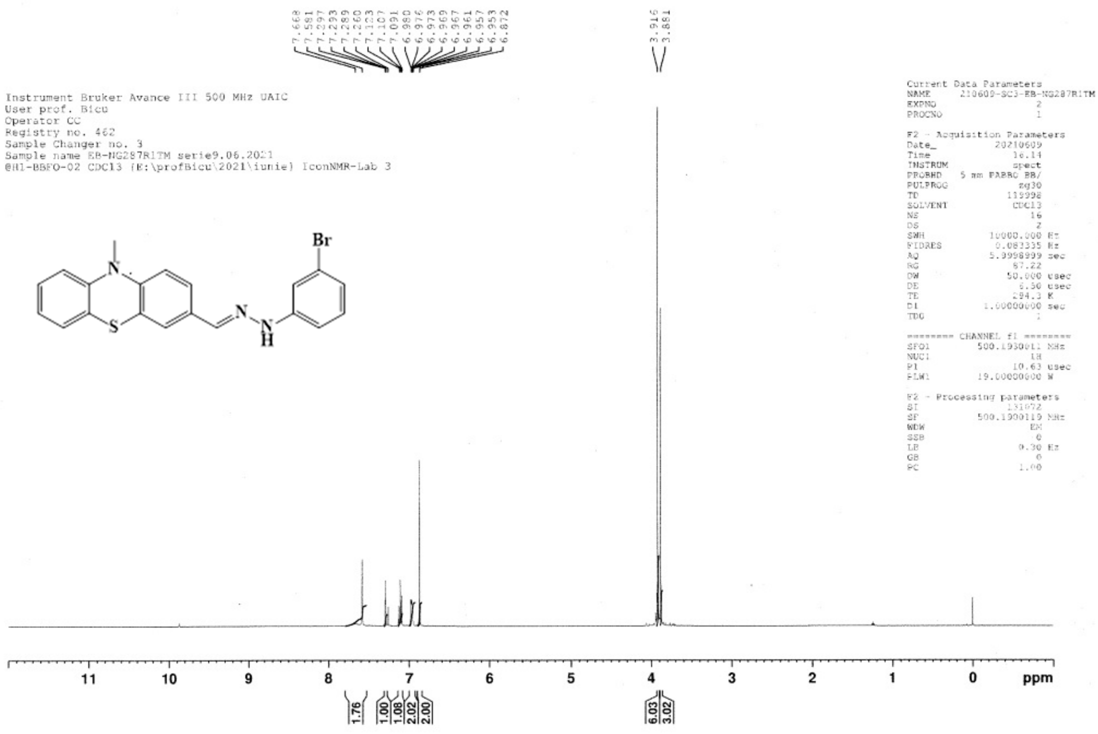

(a)

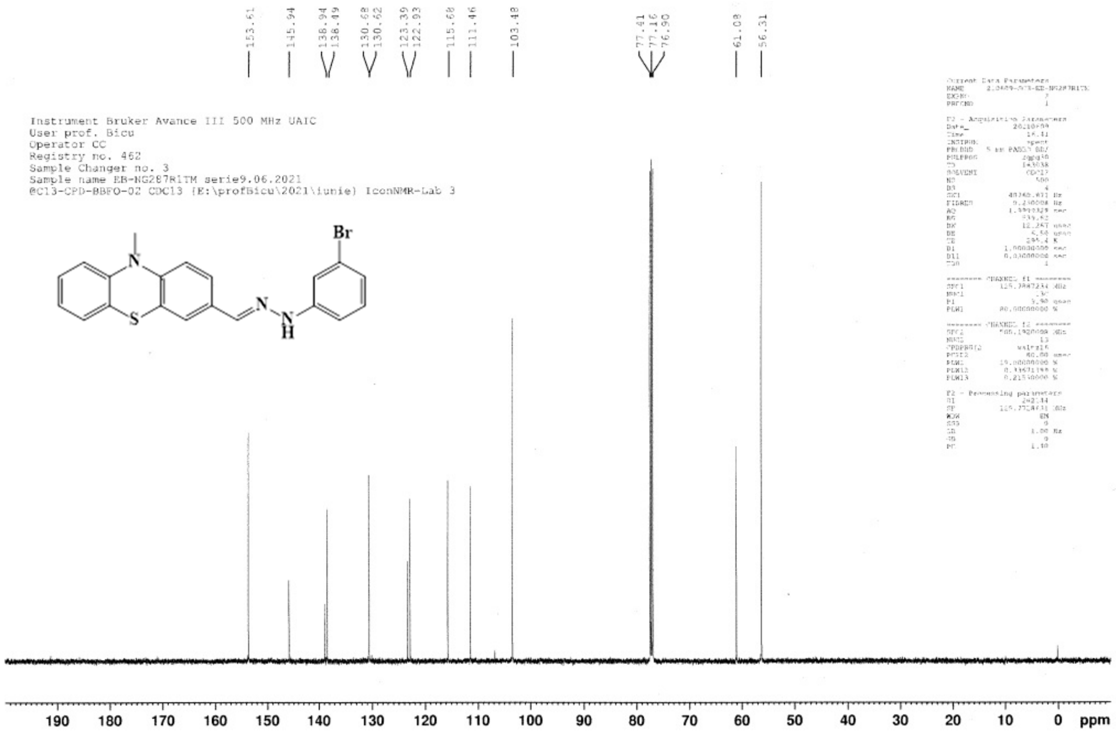

(b)

Figure S12. (a) ^{1}H-NMR and (b) ^{13}C-NMR spectra for hydrazone **12**

4-(2-((1*H*-indol-3-yl)methylene)hydrazinyl)-7-chloroquinoline (**13**).

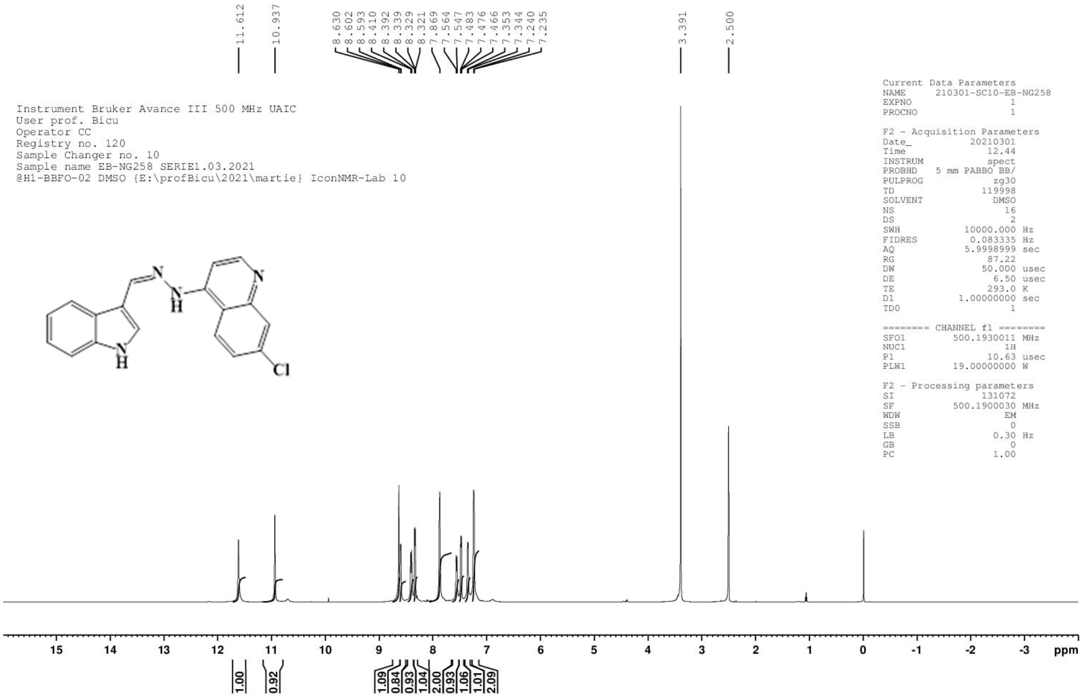

(a)

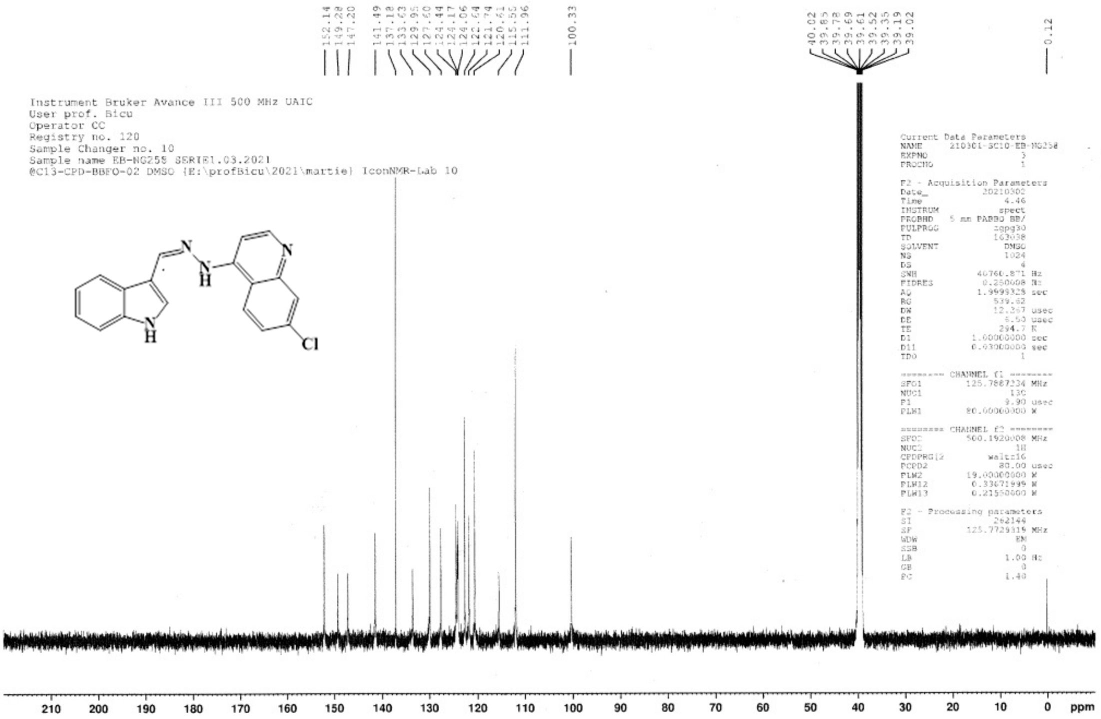

(b)

Figure S13. (a) ^{1}H-NMR and (b) ^{13}C-NMR spectra for hydrazone **13**

3-((2-(6-chloropyridin-2-yl)hydrazono)methyl)-1*H*-indole (**14**).

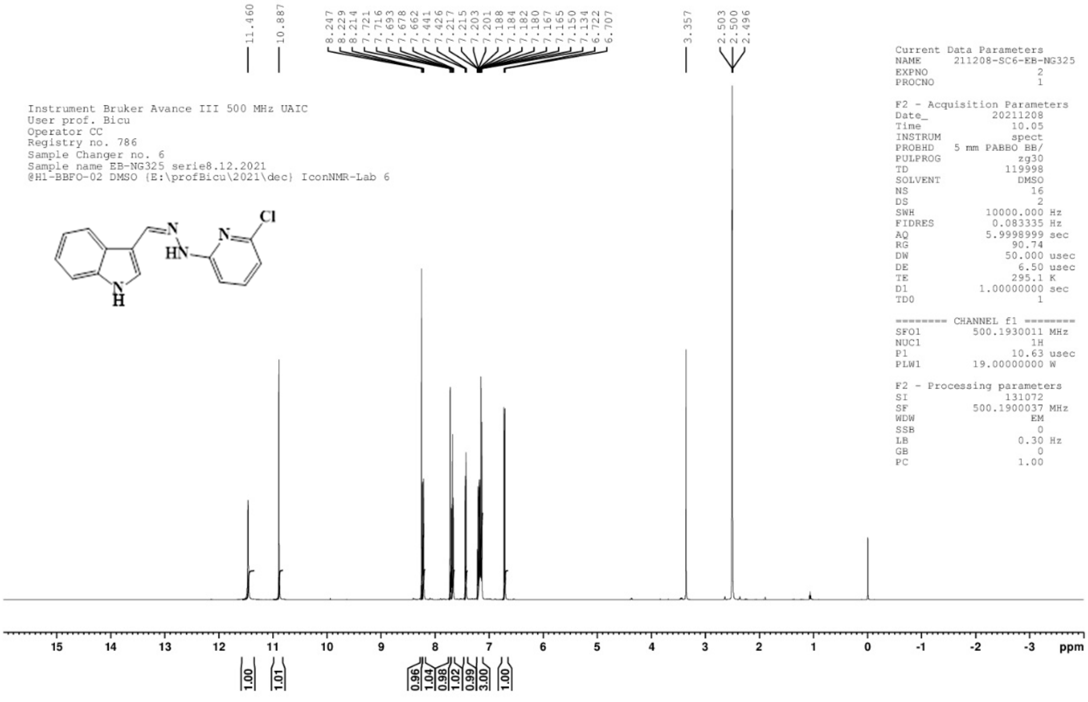

(a)

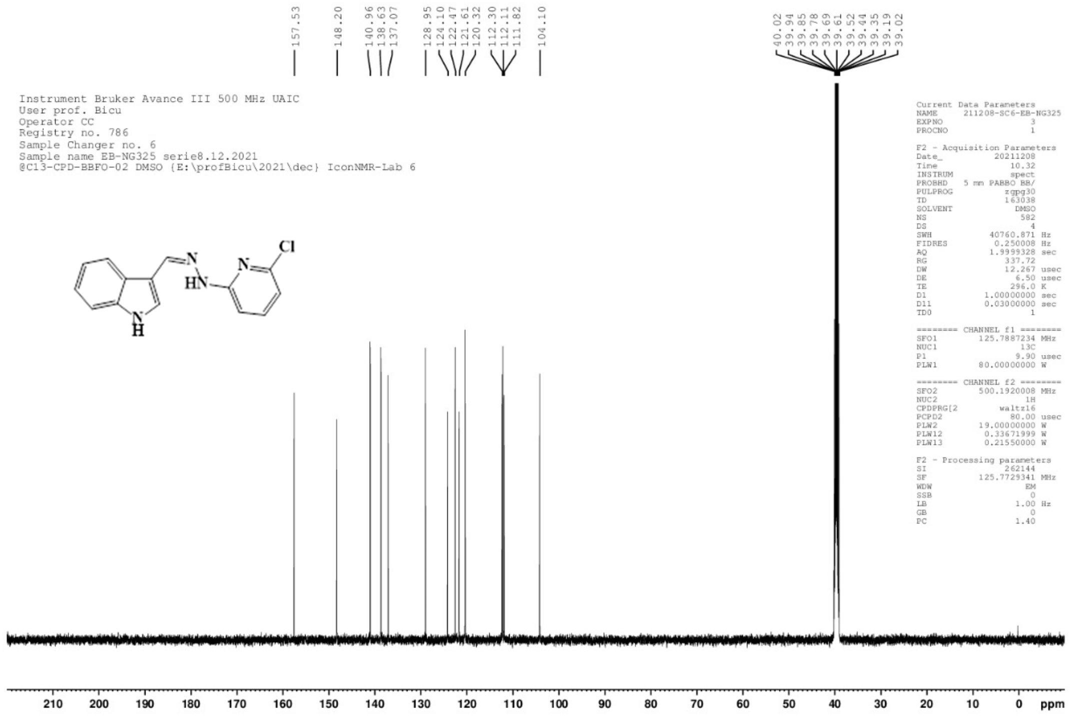

(b)

Figure S14. (a) ^{1}H-NMR and (b) ^{13}C-NMR spectra for hydrazone **14**

3-((2-(5-bromopyridin-2-yl)hydrazono)methyl)-1*H*-indole (**15**).

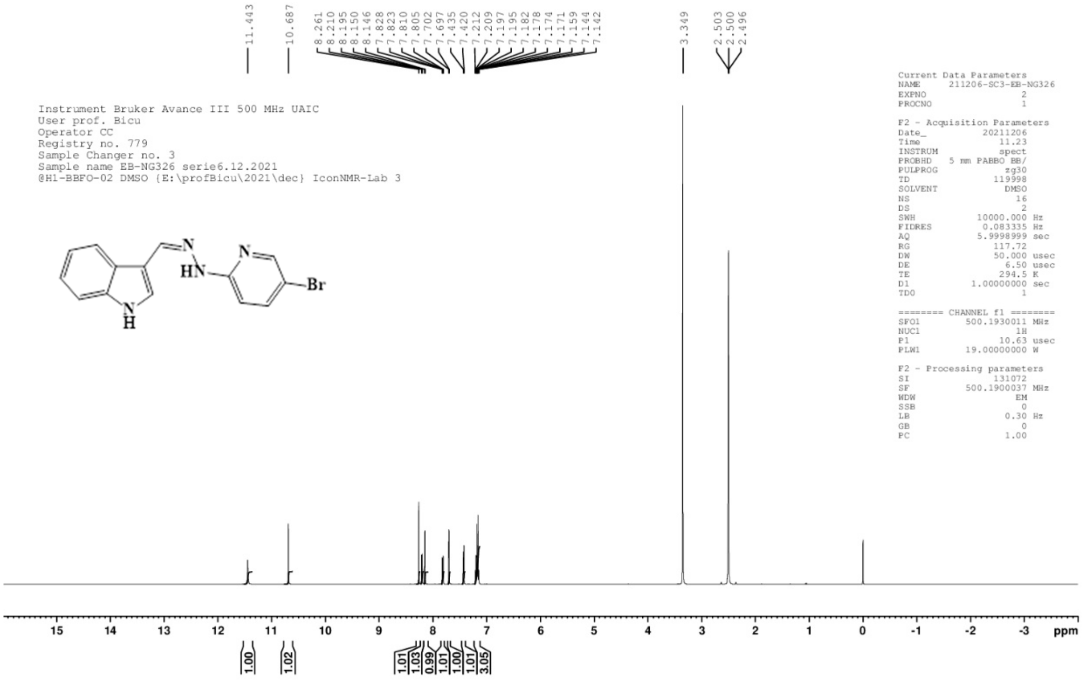

(a)

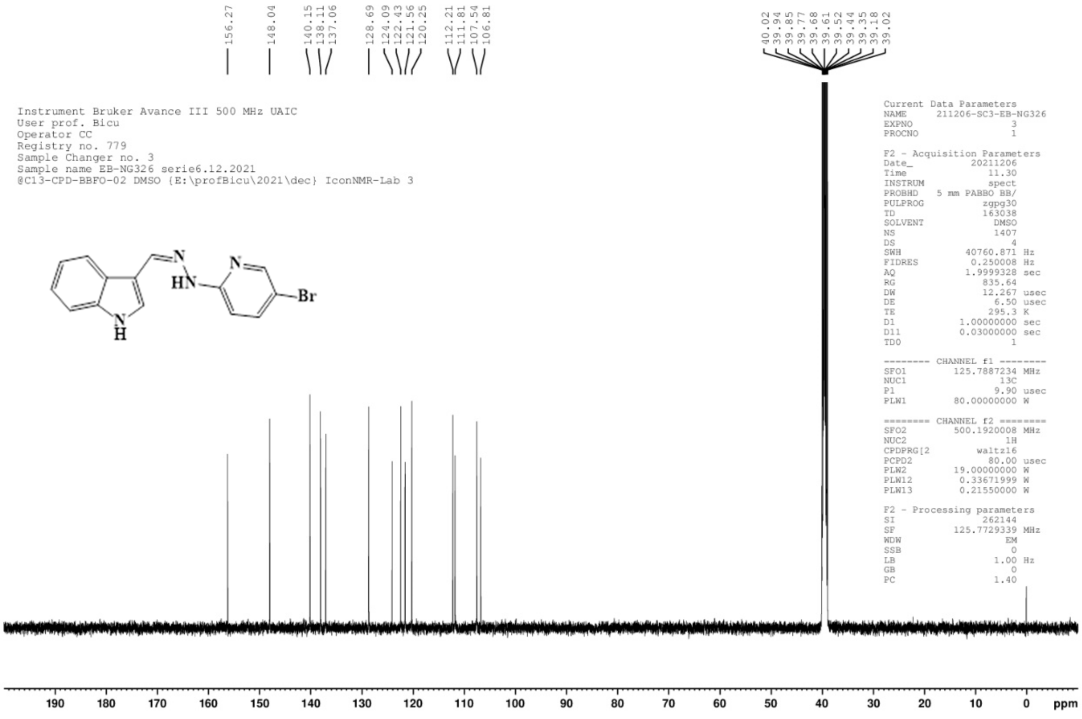

(b)

Figure S15. (a) ^{1}H-NMR and (b) ^{13}C-NMR spectra for hydrazone **15**

7-chloro-4-(2-((5-methoxy-1*H*-indol-3-yl)methylene)hydrazinyl)quinoline (**16**).

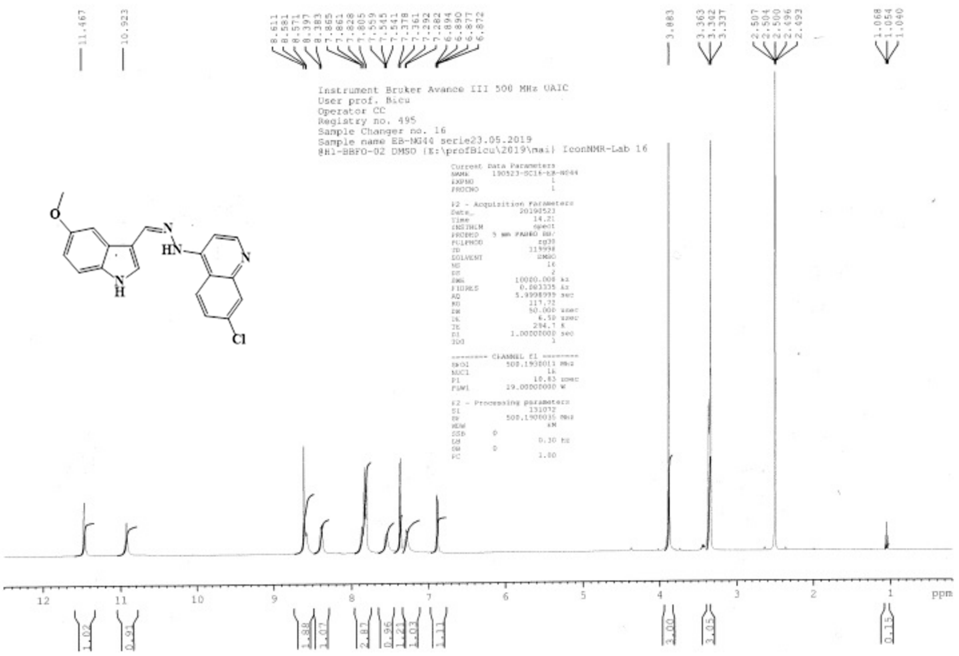

(a)

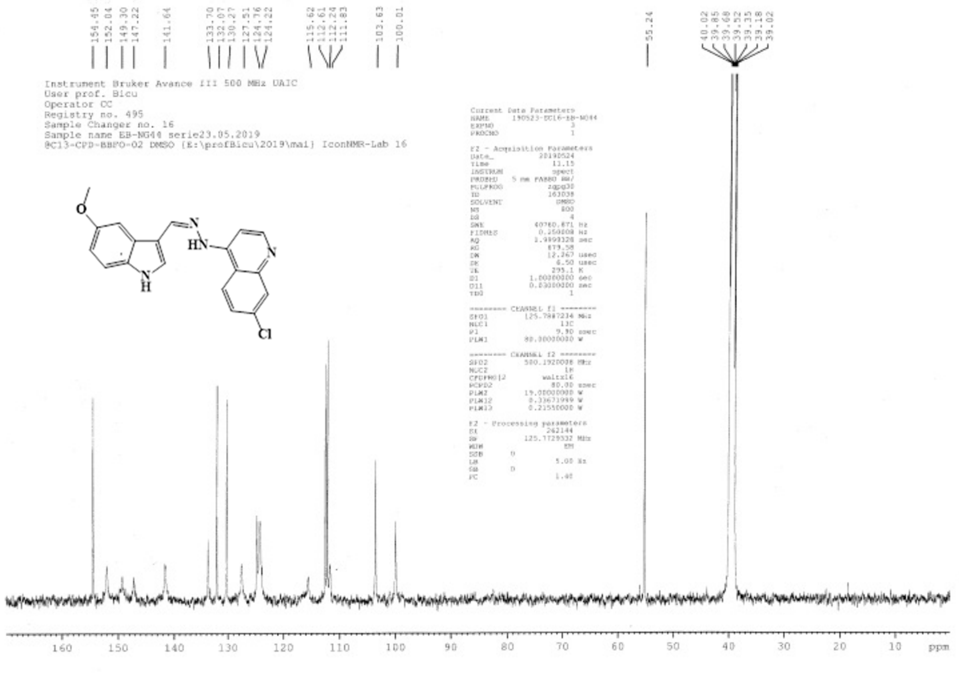

(b)

Figure S16. (a) ^{1}H-NMR and (b) ^{13}C-NMR spectra for hydrazone **16**

2-(2-((5-methoxy-1*H*-indol-3-yl)methylene)hydrazinyl)benzo[*d*]thiazole (**17**).

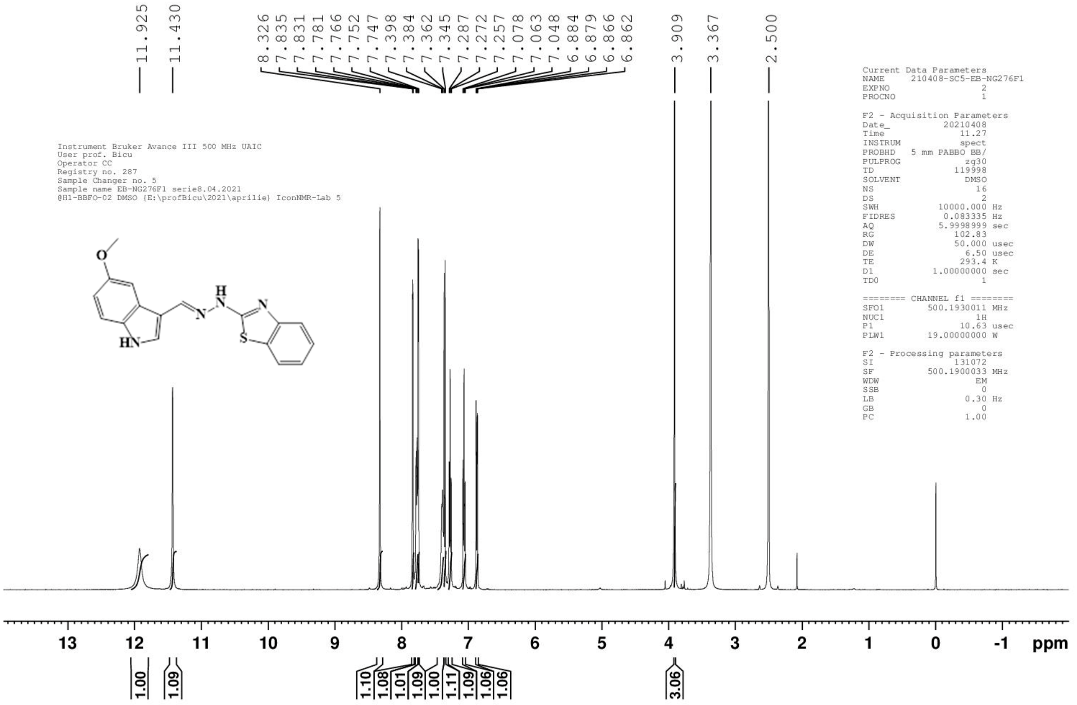

(a)

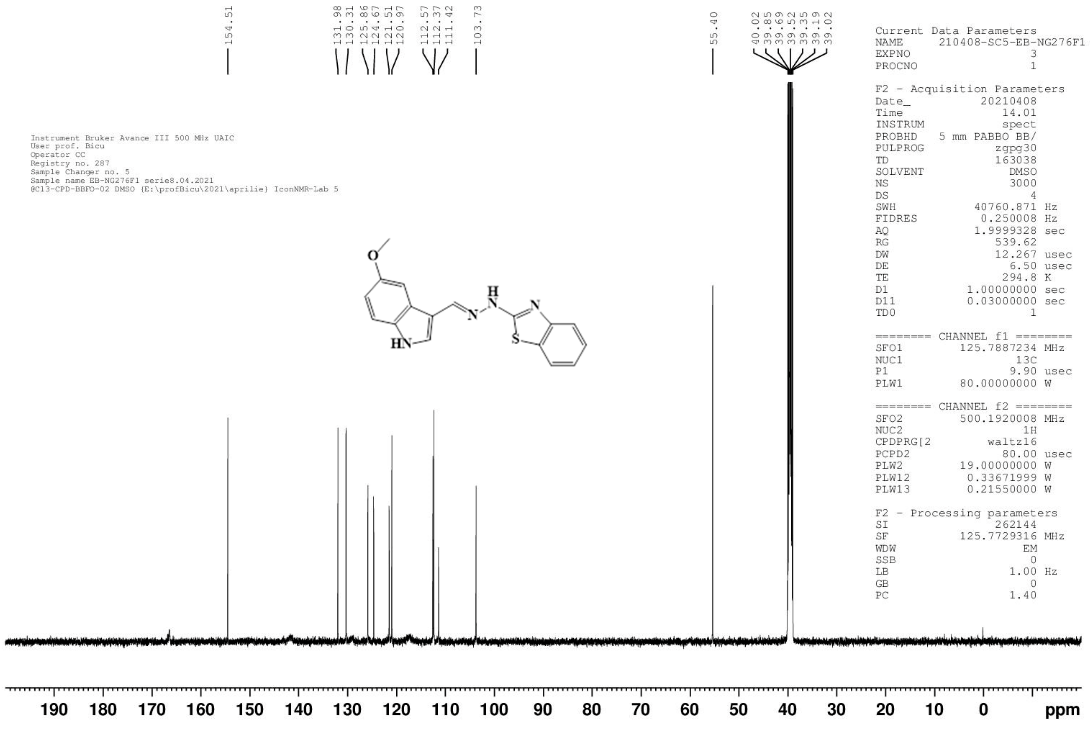

(b)

Figure S17. (a) ^{1}H-NMR and (b) ^{13}C-NMR spectra for hydrazone **17**

3-((2-(5-bromopyridin-2-yl)hydrazono)methyl)-5-methoxy-1*H*-indole (**18**).

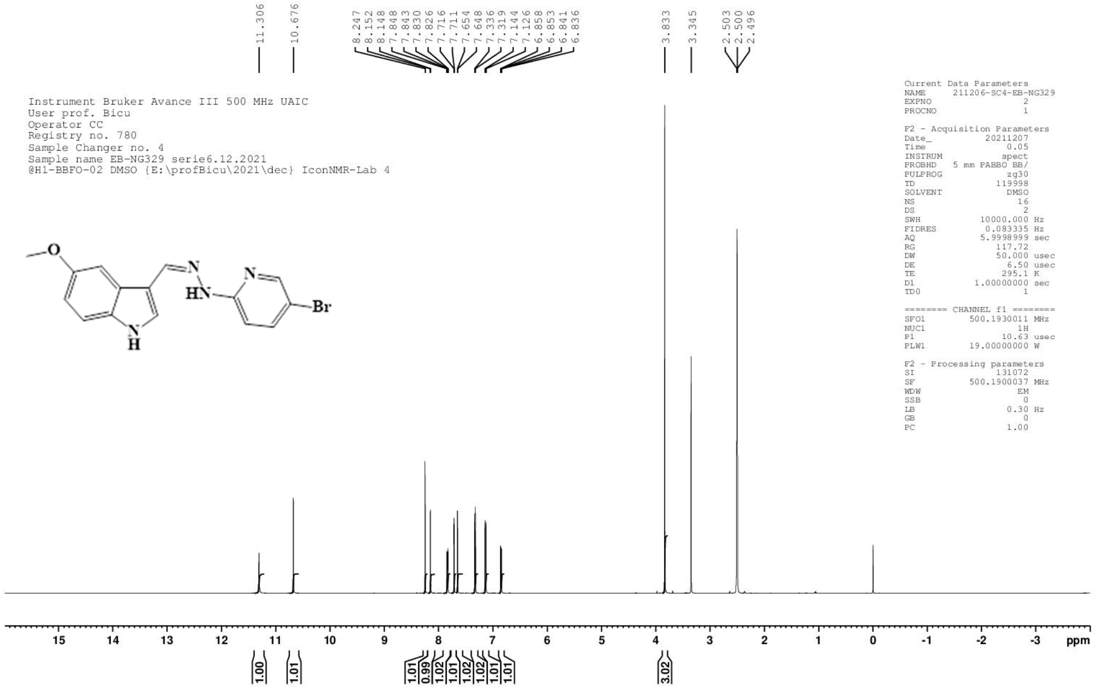

(a)

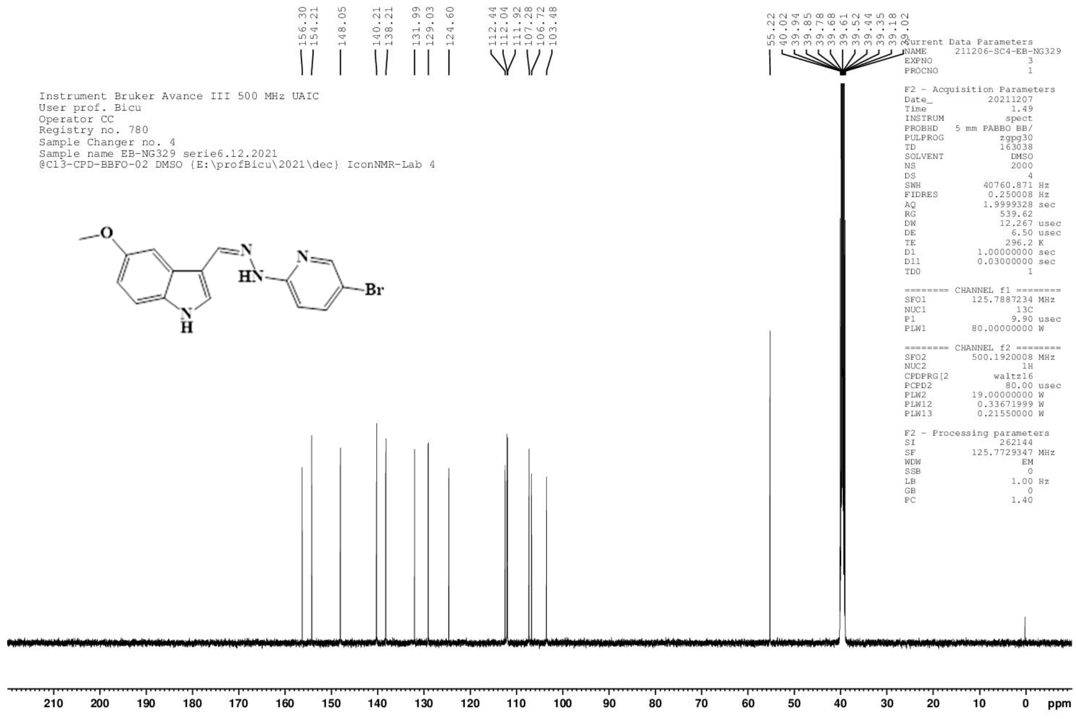

(b)

Figure S18. (a) ^{1}H-NMR and (b) ^{13}C-NMR spectra for hydrazone **18**

3-((2-(6-chloropyridin-2-yl)hydrazono)methyl)-5-methoxy-1*H*-indole (**19**).

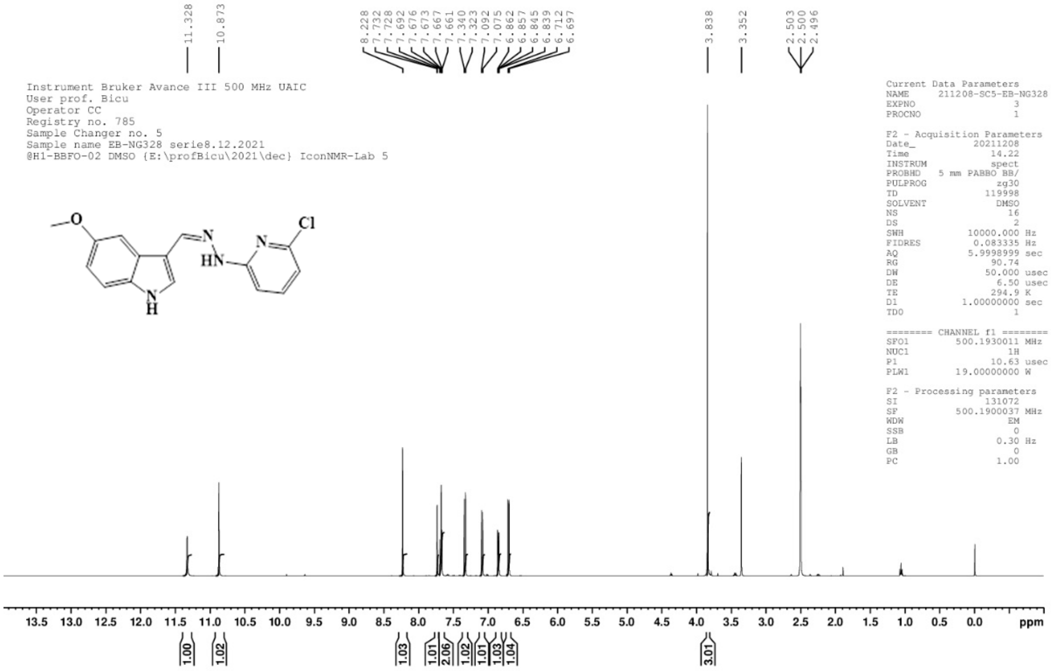

(a)

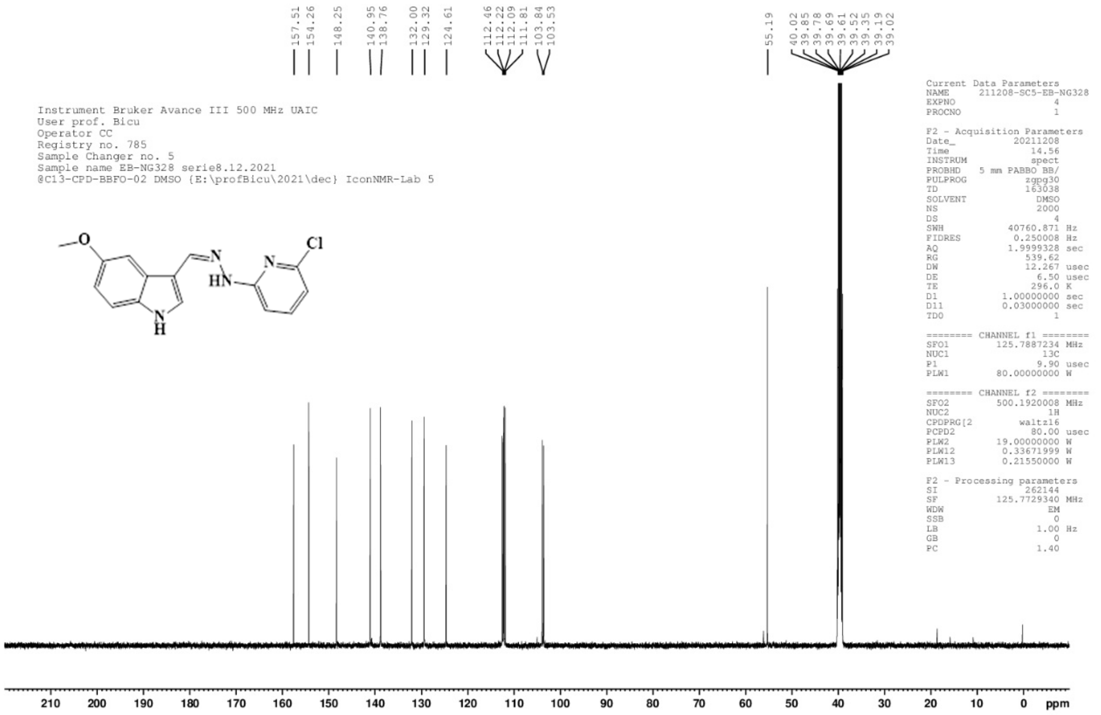

(b)

Figure S19. (a) ^{1}H-NMR and (b) ^{13}C-NMR spectra for hydrazone **19**

7-chloro-4-(2-((1-methyl-1*H*-indol-3-yl)methylene)hydrazinyl)quinoline (**20**).

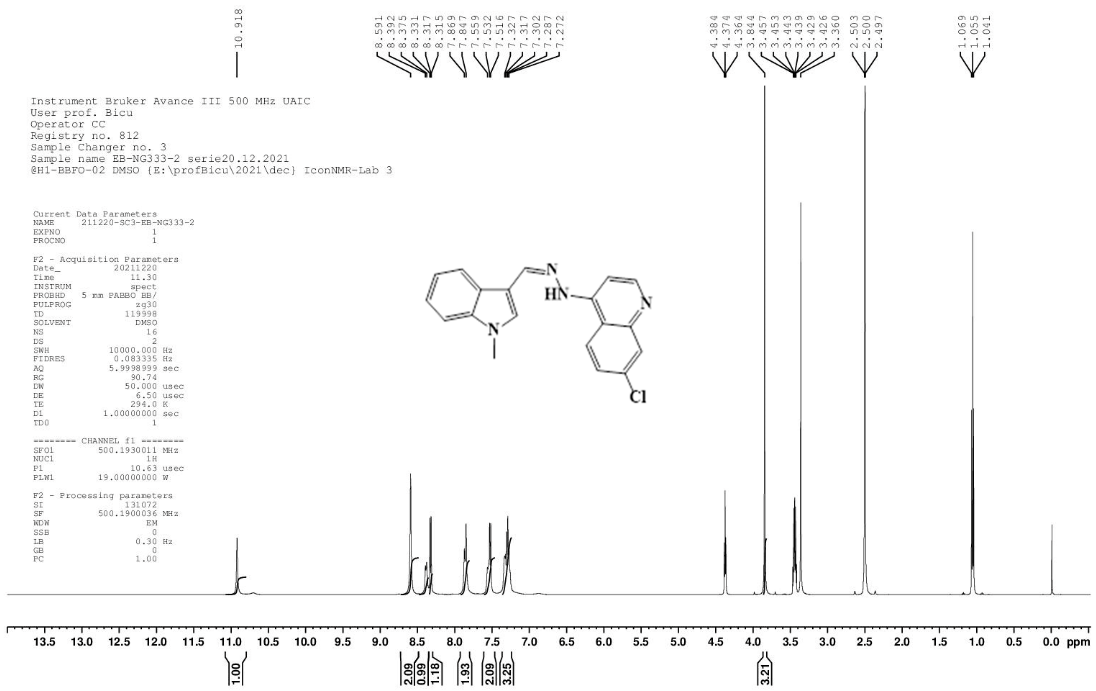

(a)

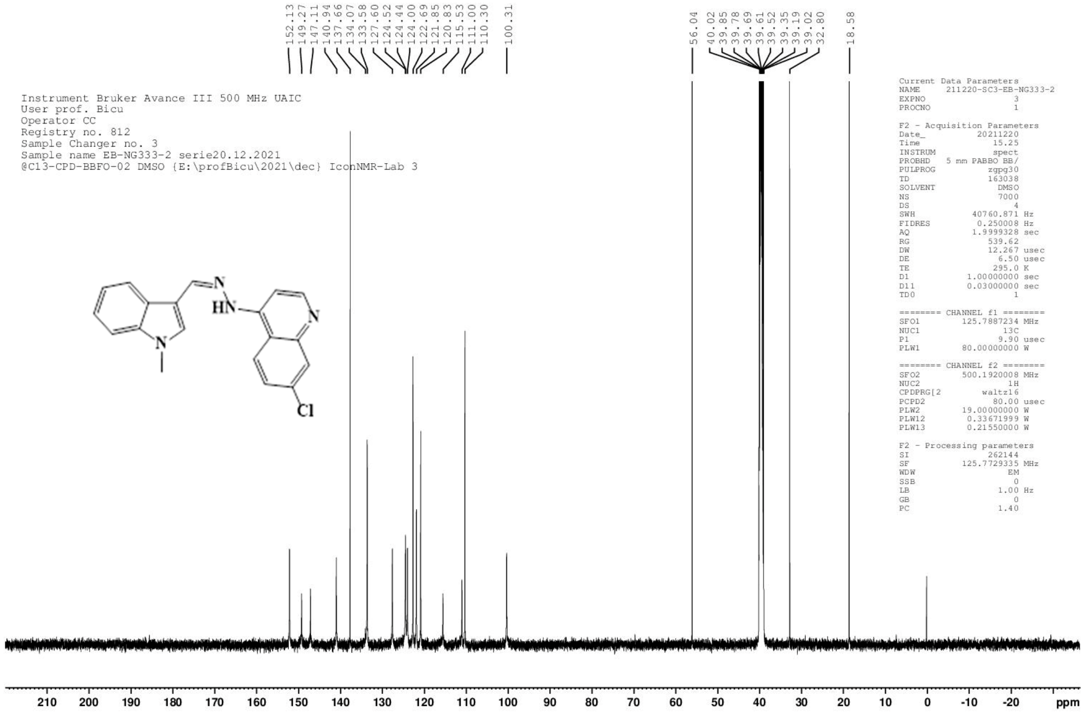

(b)

Figure S20. (a) ^{1}H-NMR and (b) ^{13}C-NMR spectra for hydrazone **20**

3-((2-(5-bromopyridin-2-yl)hydrazono)methyl)-1-methyl-1*H*-indole (**21**).

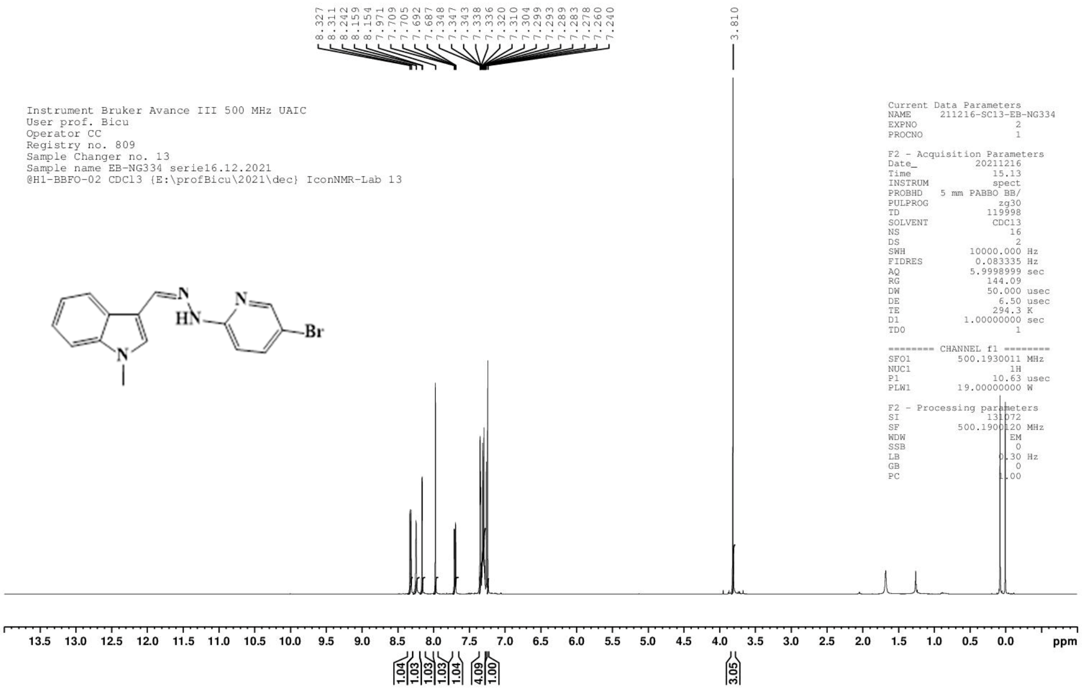

(a)

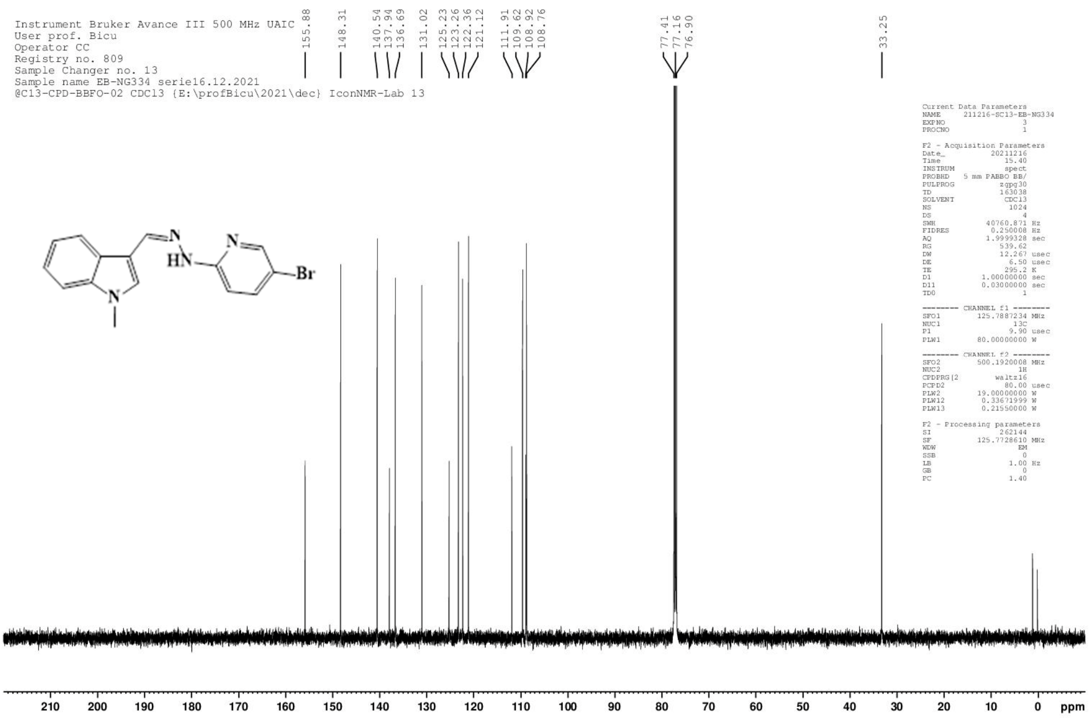

(b)

Figure S21. (a) ^{1}H-NMR and (b) ^{13}C-NMR spectra for hydrazone **21**

3-((2-(6-chloropyridin-2-yl)hydrazono)methyl)-1-methyl-1*H*-indole (**22**).

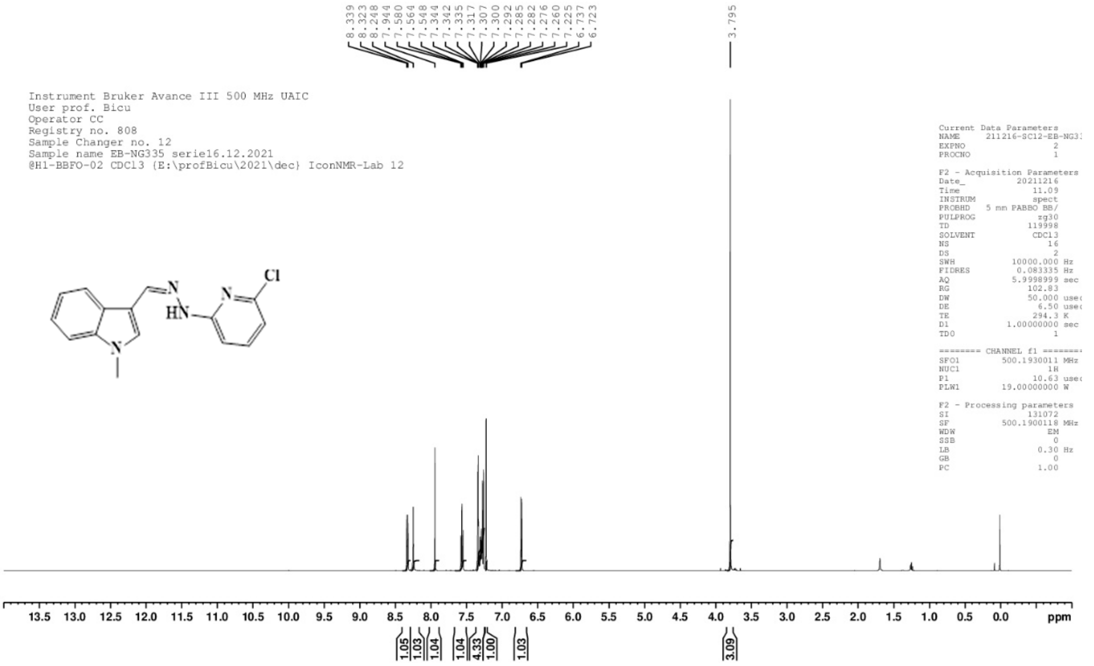

(a)

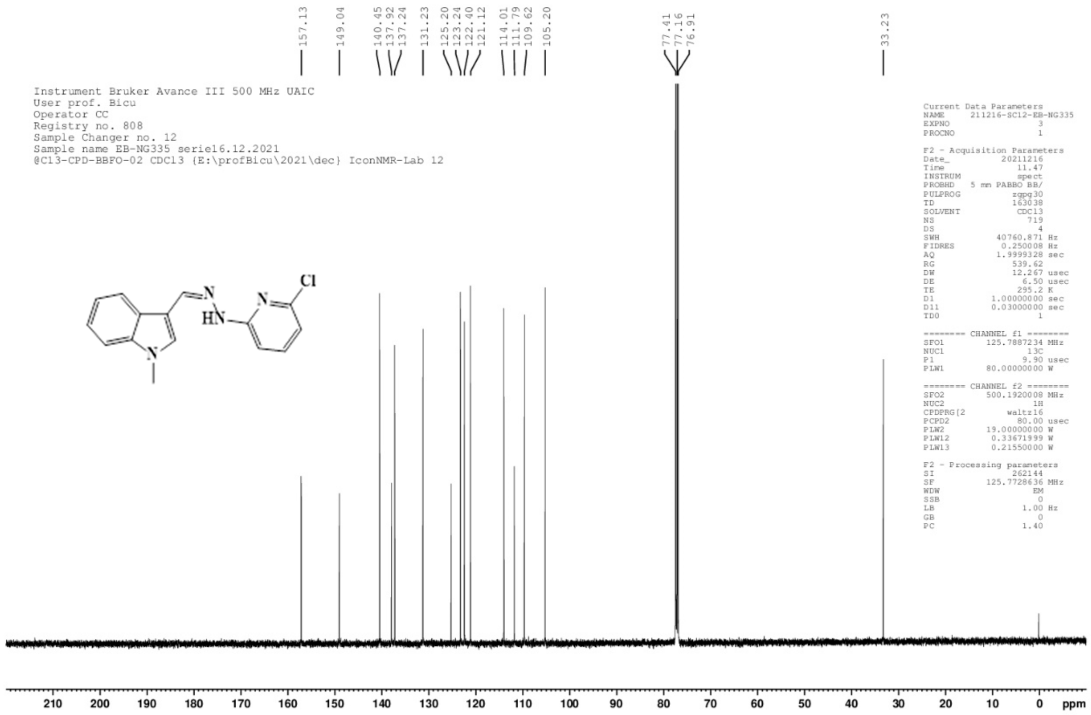

(b)

Figure S22. (a) ^{1}H-NMR and (b) ^{13}C-NMR spectra for hydrazone **22**

7-chloro-4-(2-((5-methoxy-1-methyl-1*H*-indol-3-yl)methylene)hydrazinyl)quinoline (**23**).

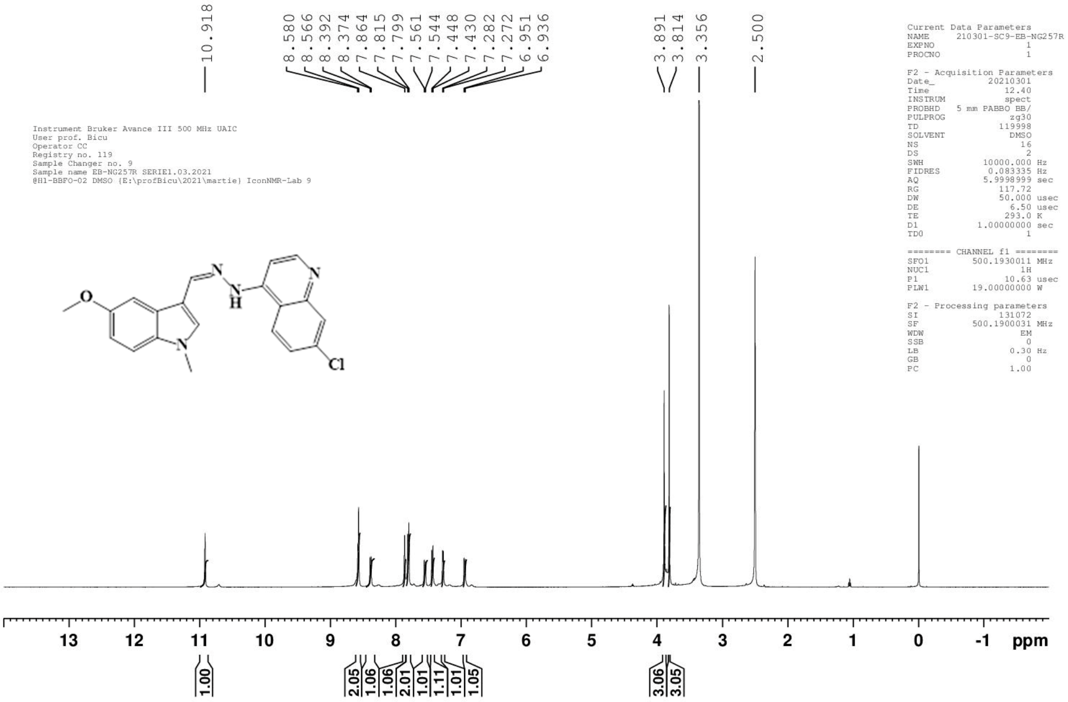

(a)

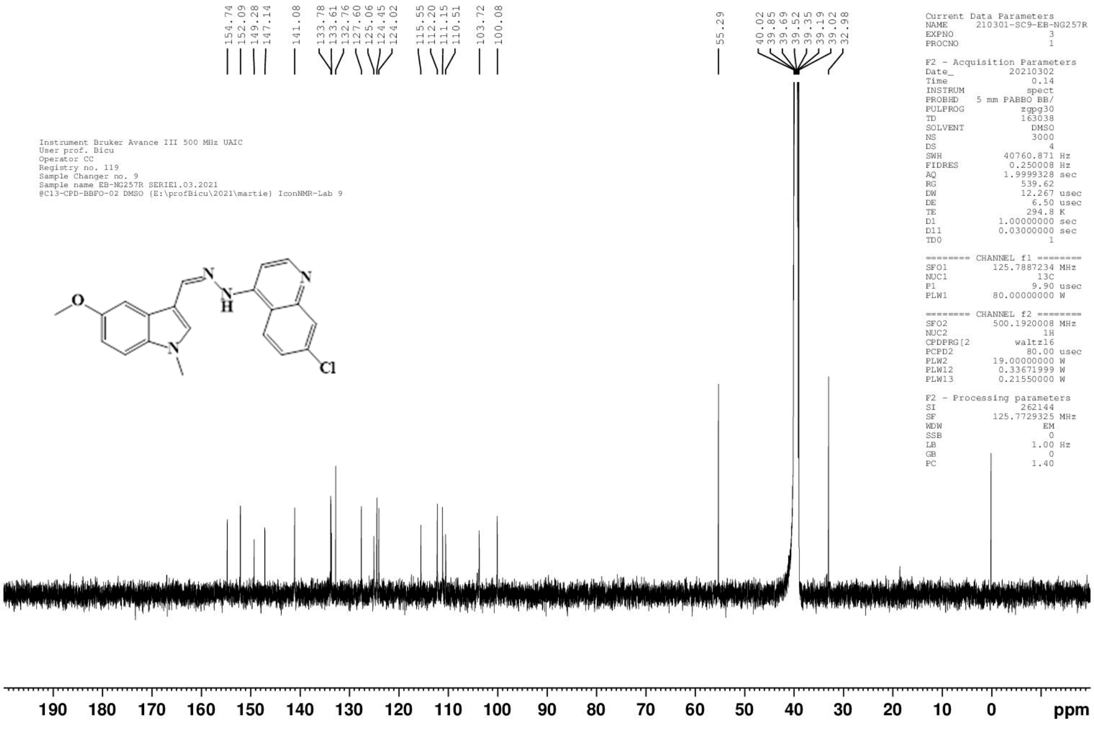

(b)

Figure S23. (a) ^{1}H-NMR and (b) ^{13}C-NMR spectra for hydrazone **23**

2-(2-((5-methoxy-1-methyl-1*H*-indol-3-yl)methylene)hydrazinyl)quinoline (**24**).

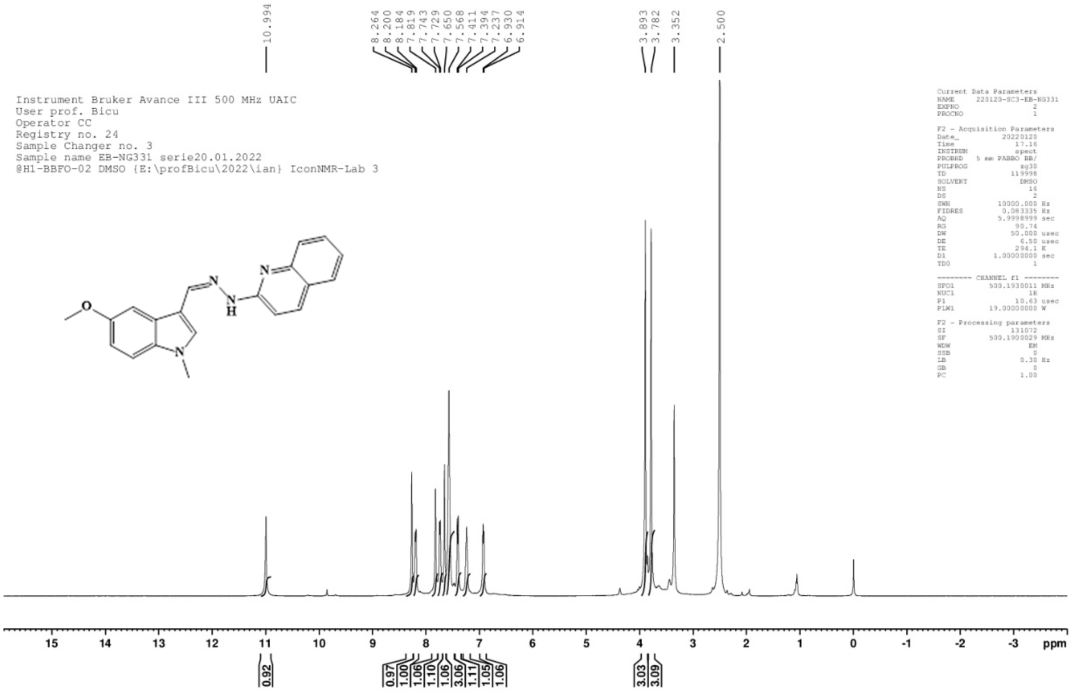

(a)

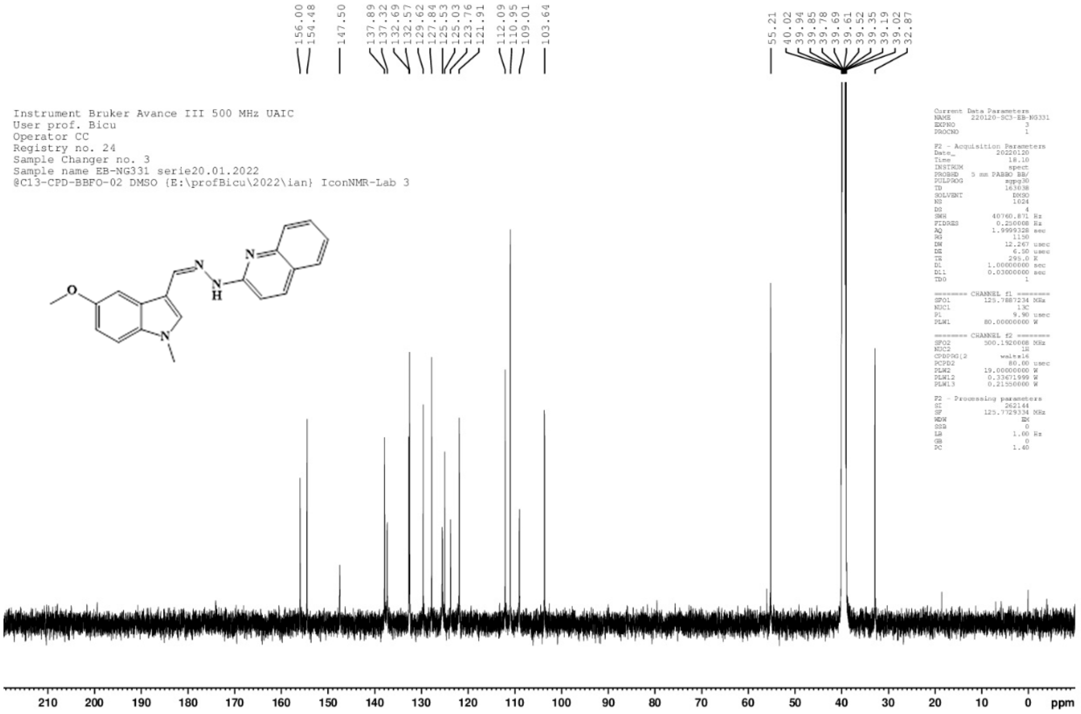

(b)

Figure S24. (a) ^{1}H-NMR and (b) ^{13}C-NMR spectra for hydrazone **24**

4-(2-((5-bromo-1*H*-indol-3-yl)methylene)hydrazinyl)-7-chloroquinoline (**25**).

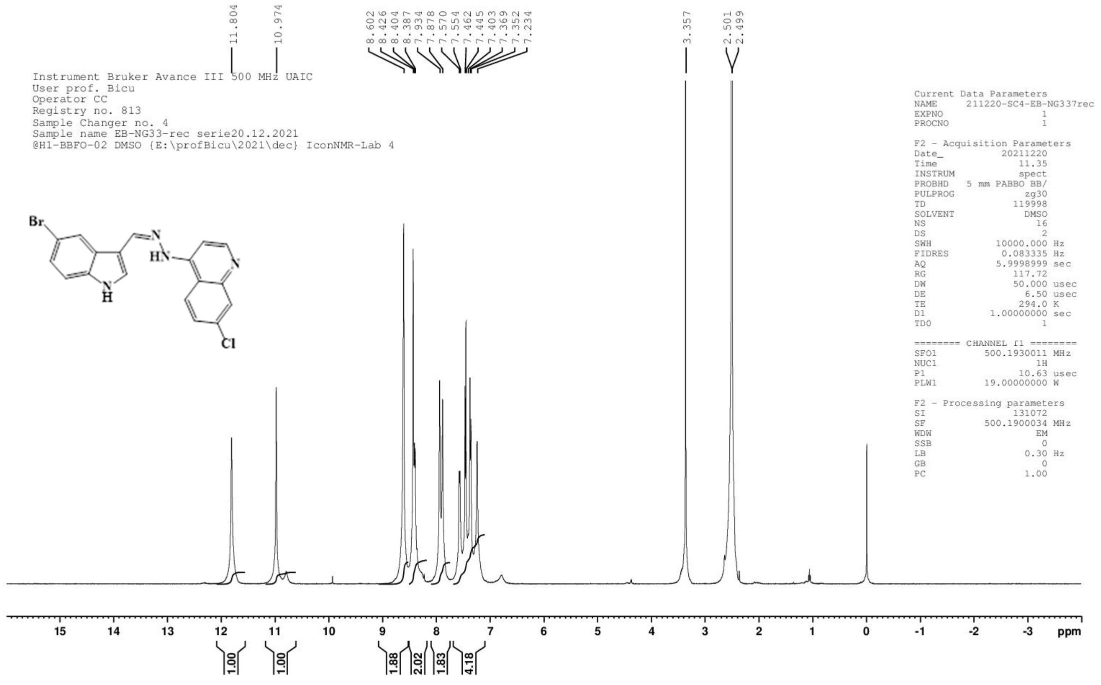

(a)

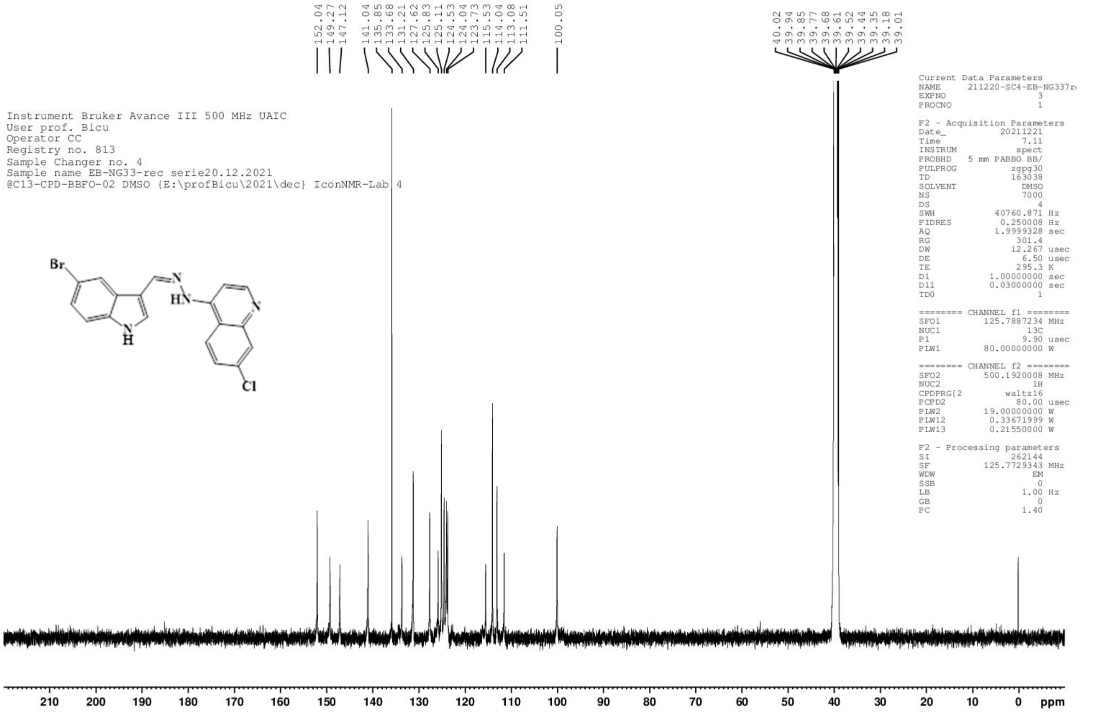

(b)

Figure S25. (a) ^{1}H-NMR and (b) ^{13}C-NMR spectra for hydrazone **25**

5-bromo-3-((2-(5-bromopyridin-2-yl)hydrazono)methyl)-1*H*-indole (**26**).

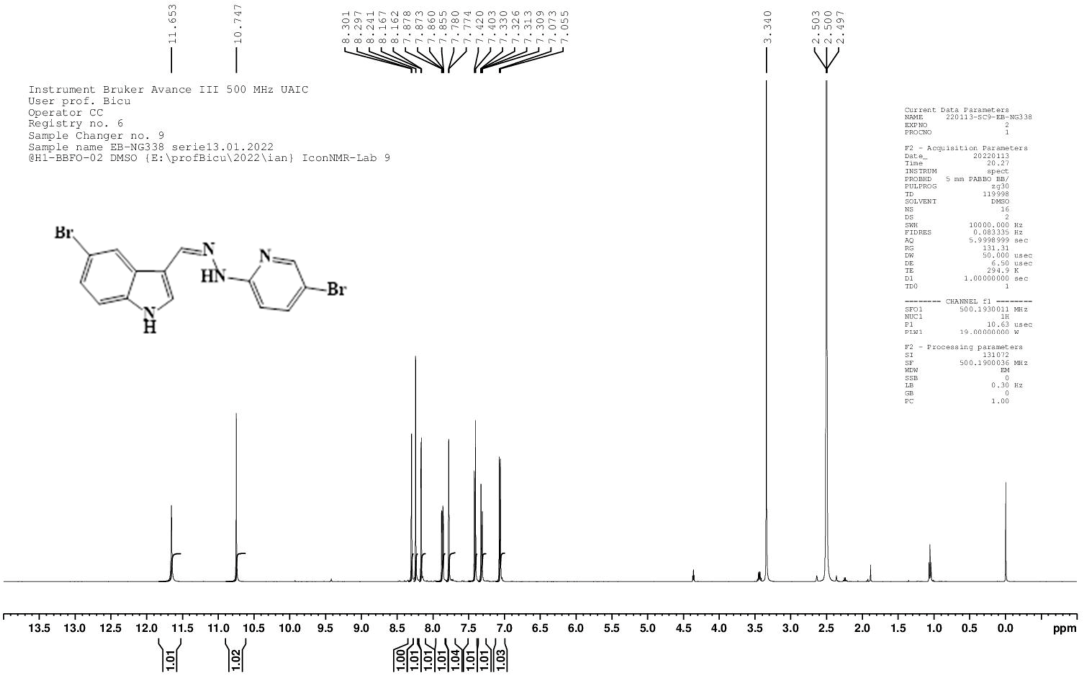

(a)

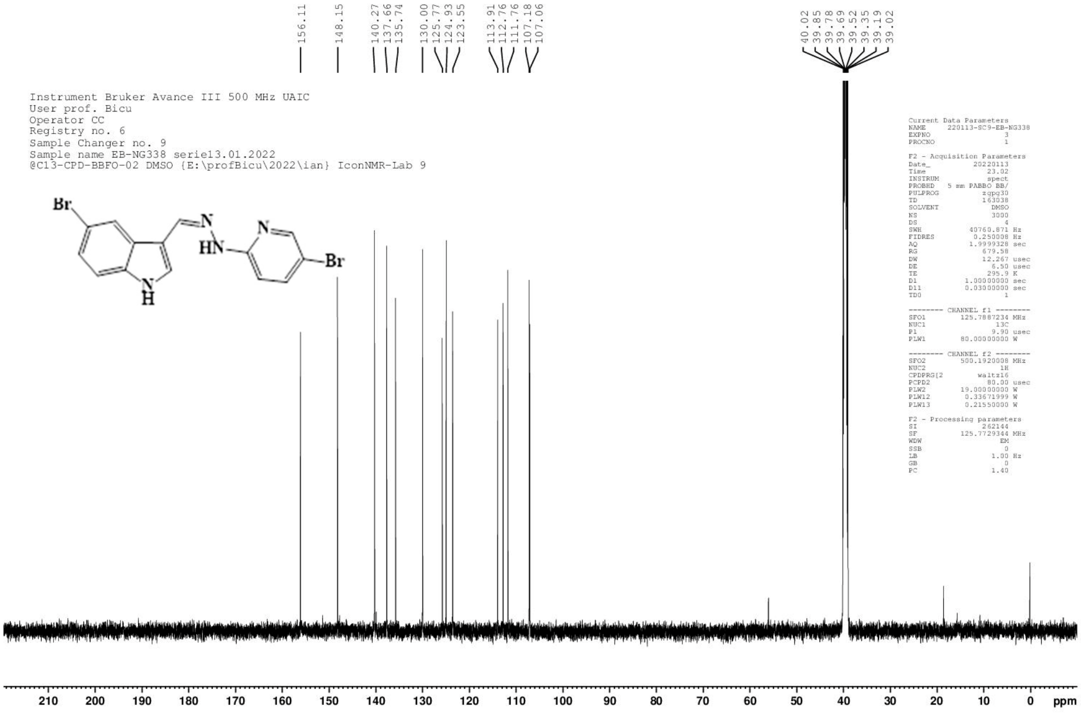

(b)

Figure S26. (a) ^{1}H-NMR and (b) ^{13}C-NMR spectra for hydrazone **26**

5-bromo-3-((2-(6-chloropyridin-2-yl)hydrazono)methyl)-1*H*-indole (**27**).

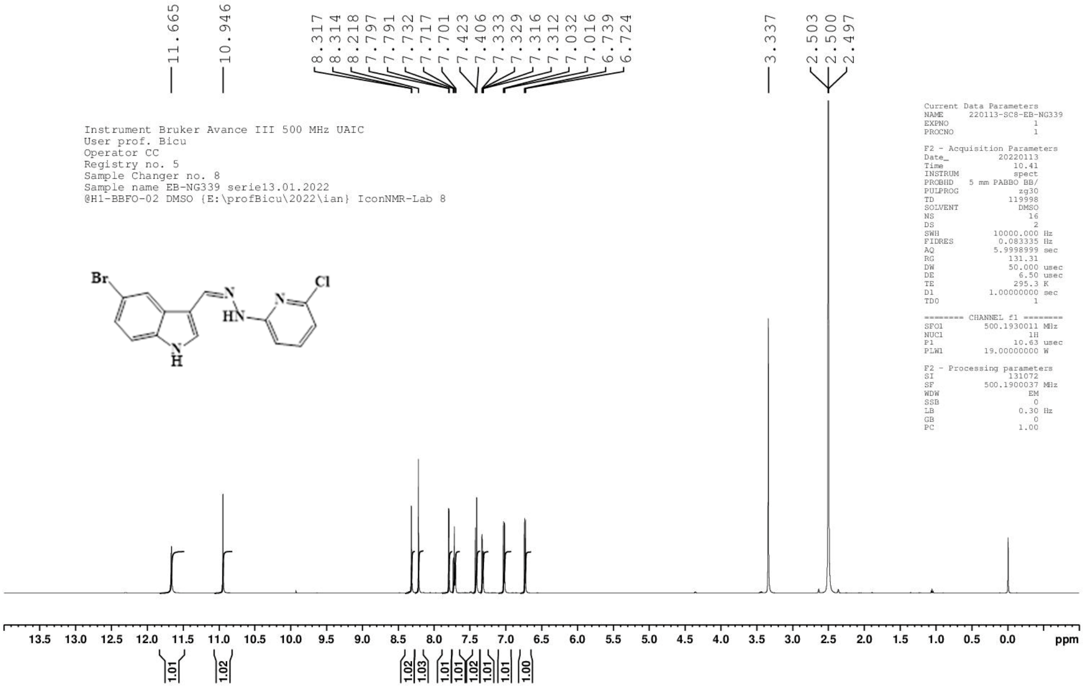

(a)

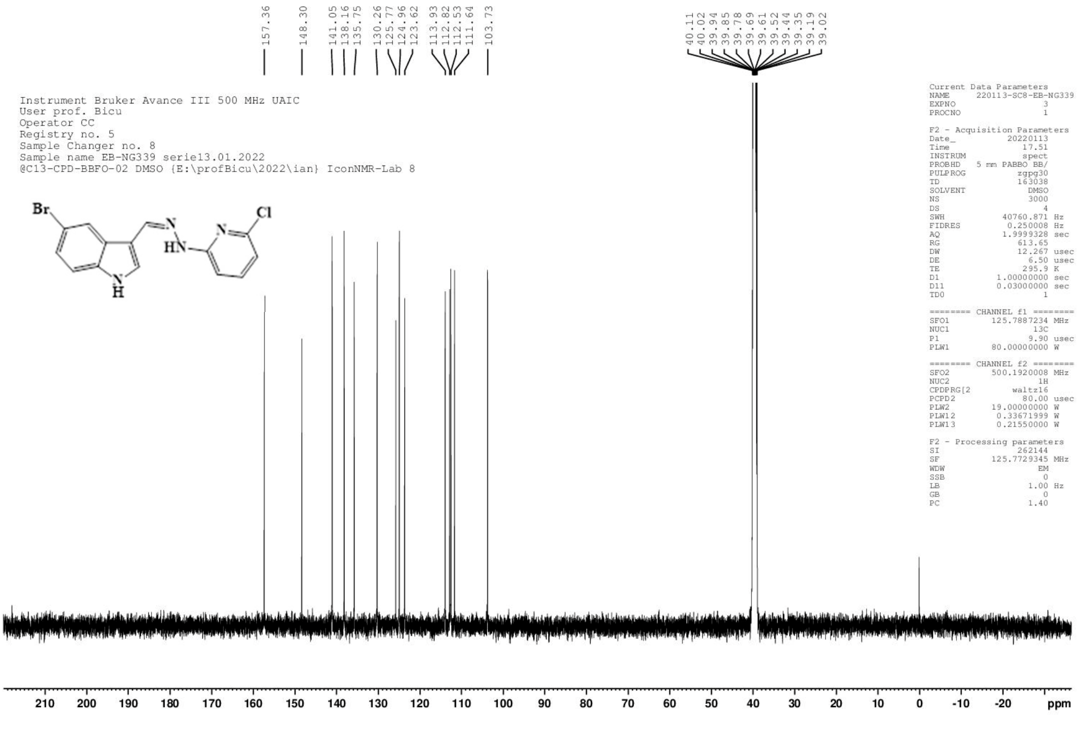

(b)

Figure S27. (a) ^{1}H-NMR and (b) ^{13}C-NMR spectra for hydrazone **27**
